# Neuroimaging Characterization of Acute Traumatic Brain Injury with Focus on Frontline Clinicians: Recommendations from the 2024 National Institute of Neurological Disorders and Stroke Traumatic Brain Injury Classification and Nomenclature Initiative Imaging Working Group

**DOI:** 10.1089/neu.2025.0079

**Published:** 2025-07-09

**Authors:** Christine L. Mac Donald, Esther L. Yuh, Thijs Vande Vyvere, Brian L. Edlow, Lucia M. Li, Andrew R. Mayer, Pratik Mukherjee, Virginia F.J. Newcombe, Elisabeth A. Wilde, Inga K. Koerte, Deborah Yurgelun-Todd, Yu-Chien Wu, Ann-Christine Duhaime, Hibah O. Awwad, Kristen Dams-O’Connor, Adele Doperalski, Andrew I.R. Maas, Michael A. McCrea, Nsini Umoh, Geoffrey T. Manley

**Affiliations:** ^1^ Department of Neurological Surgery, University of Washington School of Medicine, Seattle, Washington, USA.; ^2^ Department of Radiology, University of California, San Francisco, San Francisco, California, USA.; ^3^ Department of Radiology, Antwerp University Hospital, Antwerp, Belgium.; ^4^ Department of Neurology, Center for Neurotechnology and Neurorecovery, Massachusetts General Hospital and Harvard Medical School, Boston, Massachusetts, USA.; ^5^ Athinoula A. Martinos Center for Biomedical Imaging, Massachusetts General Hospital, Charlestown, Massachusetts, USA.; ^6^ Centre for Health Care and Technology, Imperial College London, London, United Kingdom.; ^7^ The Mind Research Network, Albuquerque, New Mexico, USA.; ^8^ Department of Medicine, University of Cambridge, Cambridge, United Kingdom.; ^9^ Department of Neurology, University of Utah, Salt Lake City, Utah, USA.; ^10^ George E. Wahlen VA Salt Lake City Healthcare System, Salt Lake City, Utah, USA.; ^11^ Department of Child and Adolescent Psychiatry, Ludwig-Maximilians-Universität, Munich, Germany.; ^12^ Department of Psychiatry, Salt Lake City VA MIRECC, University of Utah, Salt Lake City, Utah, USA.; ^13^ Department of Radiology and Imaging Sciences, Indiana University School of Medicine, Indianapolis, Indiana, USA.; ^14^ Department of Neurosurgery, Massachusetts General Hospital, Boston, Massachusetts, USA.; ^15^ Division of Neuroscience, National Institute of Neurological Disorders and Stroke, Bethesda, Maryland, USA.; ^16^ Department of Rehabilitation and Human Performance, Icahn School of Medicine, Mount Sinai, New York, New York, USA.; ^17^ Department of Neurology, Icahn School of Medicine, Mount Sinai, New York, New York, USA.; ^18^ Department of Neurosurgery, Antwerp University Hospital, Edegem, Belgium.; ^19^ Department of Translational Neuroscience, Faculty of Medicine and Health Science, University of Antwerp, Antwerp, Belgium.; ^20^ Department of Neurosurgery, Medical College of Wisconsin, Milwaukee, Wisconsin, USA.; ^21^ Department Neurological Surgery, University of California San Francisco, San Francisco, California, USA.

**Keywords:** brain imaging, CBI-M model, classification, CT, nomenclature, TBI

## Abstract

Neuroimaging screening and surveillance is one of the first frontline diagnostic tools leveraged in the acute assessment (first 24 h postinjury) of patients suspected to have traumatic brain injury (TBI). While imaging, in particular computed tomography, is used almost universally in emergency departments worldwide to evaluate possible features of TBI, there is no currently agreed-upon reporting system, standard terminology, or framework to contextualize brain imaging findings with other available medical, psychosocial, and environmental data. In 2023, the NIH-National Institute of Neurological Disorders and Stroke convened six working groups of international experts in TBI to develop a new framework for nomenclature and classification. The goal of this effort was to propose a more granular system of injury classification that incorporates recent progress in imaging biomarkers, blood-based biomarkers, and injury and recovery modifiers to replace the commonly used Glasgow Coma Scale-based diagnosis groups of mild, moderate, and severe TBI, which have shown relatively poor diagnostic, prognostic, and therapeutic utility. Motivated by prior efforts to standardize the nomenclature for pathoanatomic imaging findings of TBI for research and clinical trials, along with more recent studies supporting the refinement of the originally proposed definitions, the Imaging Working Group sought to update and expand this application specifically for consideration of use in clinical practice. Here we report the recommendations of this working group to enable the translation of structured imaging common data elements to the standard of care. These leverage recent advances in imaging technology, electronic medical record (EMR) systems, and artificial intelligence (AI), along with input from key stakeholders, including patients with lived experience, caretakers, providers across medical disciplines, radiology industry partners, and policymakers. It was recommended that (1) there would be updates to the definitions of key imaging features used for this system of classification and that these should be further refined as new evidence of the underlying pathology driving the signal change is identified; (2) there would be an efficient, integrated tool embedded in the EMR imaging reporting system developed in collaboration with industry partners; (3) this would include AI-generated evidence-based feature clusters with diagnostic, prognostic, and therapeutic implications; and (4) a “patient translator” would be developed in parallel to assist patients and families in understanding these imaging features. In addition, important disclaimers would be provided regarding known limitations of current technology until such time as they are overcome, such as resolution and sequence parameter considerations. The end goal is a multifaceted TBI characterization model incorporating clinical, imaging, blood biomarker, and psychosocial and environmental modifiers to better serve patients not only acutely but also through the postinjury continuum in the days, months, and years that follow TBI.

## Introduction

In 2023, the NIH-National Institute of Neurological Disorders and Stroke (NINDS) convened six expert working groups to develop a new framework for nomenclature and classification of traumatic brain injury (TBI). The goal was for these working groups (Clinical Assessment, Blood-based Biomarkers, Neuroimaging, Psychosocial and Environmental Modifiers, Retrospective Classification, and Knowledge to Practice) to develop a granular system of acute injury classification, or, more accurately, a multidimensional characterization, to replace the widely used but rudimentary “mild, moderate, and severe” grading system and to harmonize terminology for frontline clinicians. The Imaging Working Group (WG) convened biweekly group meetings in 2023 to evaluate the current TBI imaging landscape with an extensive narrative literature review and with public feedback received from the TBI Classification and Nomenclature Workshop in January 2024. The group was motivated by recent efforts to standardize the nomenclature (lexicon) for pathoanatomic findings in TBI, as there are inconsistencies in terms and interpretations used in clinical practice to characterize imaging features of TBI. The group developed a set of recommendations and an extensive appendix to improve and extend the use of the original common data elements (CDE) for neuroimaging findings in TBI introduced in 2010.^1,2^ Since 2010, the CDE definitions have been used in a variety of research contexts across a broad spectrum of TBI severity and chronicity.^1–10^

Our working group builds upon prior neuroimaging CDE recommendations to extend their use into more widespread clinical practice by (1) incorporating new research findings regarding specific lesions to clarify ambiguities in terms from the original CDE recommendations,^11^ (2) proposing a clinical tool for accurate and efficient acute TBI classification by integration into existing electronic medical record (EMR) systems, and (3) recognizing that patterns of injury will help integrate the wide heterogeneity of imaging findings with premorbid patient factors, specific injury factors, treatment variability, and newer data such as blood-based biomarkers to more precisely characterize each patient’s injury for purposes of diagnosis, management decision-making, and prognostication. The overall goal of the Neuroimaging Working Group is to provide a more precise and consistent characterization of acute imaging findings in TBI as an element of a new proposed Clinical, Blood Biomarker, Imaging, and Modifiers framework (https://www.ninds.nih.gov/news-events/events/ninds-tbi-classification-and-nomenclature-workshop).

### Computed tomography

Because the initial neuroimaging modality for most acute TBI patients used globally remains a computed tomography (CT) scan,^12^ with some exceptions for children and in some specialized centers, the neuroimaging CDE definitions continue to be formulated to be compatible with CT while also including descriptors of lesions as seen on MRI.^13^ Noncontrast CT remains the near-universal first imaging exam in acute TBI around the world due to its high sensitivity for acute intracranial hemorrhage, exquisite delineation of bony detail, and lack of any absolute contraindication. In addition, its speed allows evaluation for intracranial pathology and craniofacial and cervical spine fractures in a single brief imaging session lasting <15 sec, a major advantage for patients with altered mental status and children and adults with polytrauma. Prior work examining CT classification systems such as the Marshall or Rotterdam CT scoring systems has shown good predictive value for outcomes such as mortality in historically moderate to severe TBI^14^; however, both have limitations when considering milder brain injuries, longer term and more comprehensive clinical outcomes, as well as when considering specific management of the broader array of pathoanatomical lesions associated with acute brain injury. Because CT angiograms or venograms of the head and neck (CTA, CTV) may also be clinically indicated early after injury, imaging findings and diagnoses relevant to these studies are also included.

### Structural MRI

Structural Magnetic Resonance Imaging (MRI) has superior sensitivity to CT for most acute pathoanatomic findings, including small subcortical and deep hemorrhages due to traumatic microvascular injuries, nonhemorrhagic axonal injuries, small brain contusions, and small extraaxial collections.^15–17^ Despite its superior sensitivity for intracranial pathology, most practice guidelines are either silent on the routine use of MRI in acute TBI, or describe a lack of sufficient evidence for its use in management decisions in acute TBI, including mild TBI.^18^ Challenges to its routine use in acute TBI include long scan times relative to CT, the need to screen patients for contraindications to MRI, limited access, and requirement for MRI-compatible life support devices for unstable or severely injured patients in the MRI scanner (which has a much smaller bore than that of CT). In clinical practice, MRI in acute TBI continues to be used mostly for patients with altered level of consciousness or neurological deficits beyond expected based on CT findings, or when it is clear that MRI can better characterize a patient’s injury as seen on the initial CT scan in a way that is judged to be relevant to acute clinical management. MRI also may be recommended for subacute or chronic mild TBI with persistent symptoms or deficits, in selected pediatric contexts, including suspected abusive head trauma, and in cases with suspected vascular compromise, as well as in other medico-legal cases to assess for intracranial injuries not detected by CT.^19,20^

## Key Updates to Definitions

### Traumatic axonal and/or microvascular injury

Historically, different terms have been used in various contexts for injuries thought to result primarily from high-magnitude angular acceleration/deceleration forces. These can be found on postmortem pathological examination (“Strich lesions^21^” or “shearing injuries”), on CT scan and on MRI. The term “diffuse axonal injury” (DAI) was first used to describe the postmortem pathological findings in nonhuman primates subjected to experimental, high magnitude, nonimpact angular deceleration (“inertial”) forces, associated with prolonged unconsciousness and widespread injury to axons in the hemispheric white matter and brainstem.^22^ The injury was modeled experimentally in order to help explain prolonged coma in human patients with high-energy injury mechanisms and prolonged unconsciousness but with minimal CT findings, who also had widespread axonal damage in a similar distribution to that seen in animal models on histopathology.^23^ The use of the term DAI later expanded to include injuries with lower levels of clinical severity but with lesions in a characteristic distribution on imaging, especially as MRI advances in both blood-sensitive and white-matter-focused sequences increased detection of very small lesions. Similarly, less widespread lesions in the white matter or gray–white junction sometimes were described as “traumatic axonal injury.” In the original neuroimaging CDEs, the term “traumatic axonal injury” was defined as 1–3 foci subcortical or deep hemorrhages and/or T2 FLAIR hyperintensities, and the term “DAI” was reserved for identification of 4 or more such foci in more than one lobe or region of the brain. Other historical terms for these findings include traumatic microbleeds, petechial hemorrhages, punctate hemorrhage, shear injury, traumatic vascular injury, and microhemorrhage.

However, more recent studies have shown that within this spectrum of injuries, trauma to vessels, leading to small hemorrhages (often called “microhemorrhages”), and small lesions to white matter may occur independently. For this reason, we recommend a key update to the neuroimaging CDE lexicon based on emerging evidence from radiological–pathological correlation studies,^11,24,25^ which support that a new term should be developed. We propose the term “traumatic axonal and/or microvascular injury” (TAMVI) to denote the constellation of imaging findings, typically in the subcortical and deeper brain regions, most often associated with inertial forces that cause high magnitude angular acceleration/deceleration of the head and shearing injury in the brain. Recent studies interrogating the underlying tissue pathology in these imaging lesions have highlighted inconsistencies or inaccuracies with some of these previously used descriptors,^11,24,26^ albeit on MRI, which may be considered a slight limitation. Nevertheless, a recent study reported that in postmortem brain tissue of TBI decedents, coregistered to MRI-confirmed TAMVI lesions, 37% had no histologically confirmed axonal injury, containing only microhemorrhage at the lesion site. Some lesions were not associated with axonal injury or microhemorrhage but were rather characterized by various types of vascular pathology, such as vascular congestion.^24,26^

Although much remains to be understood regarding these lesions that have been found to demonstrate heterogeneous histopathology and likely different focal pathophysiology, we have proposed the term TAMVI to refer to most cases previously referred to as “TAI,” “DAI,” traumatic microbleeds, petechial hemorrhages, etc., for several reasons, as follows. (1) Use of separate terms for lesions without and with blood products, while descriptively granular, is awkward and impractical for radiologists and other readers and also moves the field away from a clinically useful recognition of a characteristic *pattern* of radiological findings that can be expressed variably among patients. These lesions frequently co-occur in the same patients and may arise from substantially similar mechanical forces on the brain. Recognition and characterization of this pattern, which also may incorporate knowledge of other clinical and biomechanistic features of the overall patient context, more completely communicates the potential implications of the findings. (2) Hemorrhagic and nonhemorrhagic lesions in this constellation share many imaging features, including T2 hyperintensity, reduced diffusion on early MRI at 24–48 h, and the same characteristic locations, though as discussed above, small focal hemorrhages may or may not demonstrate associated axonal injury histopathologically. (3) Use of the term TAMVI does not require distinct terms based on the number of visible lesions. The prior cutoff of >4 lesions for DAI versus TAI, while reasonable, is somewhat arbitrary.

Additional context for the proposal of a new term may be helpful to clarify and recognize that this introduction has and likely will continue to engender controversy among those in the scientific and clinical communities. This specific injury constellation and proposals for alternative terms were a large focus of the Imaging WG and led to robust discussions and numerous suggestions for an alternative term that encompassed current scientific evidence but also addressed clinical needs. Discussions included both the focused Imaging WG as well as input during the public comment period and at the TBI Classifications Workshop in January 2024, where more than 60 experts and other key stakeholders joined the Imaging WG members. Additional participants provided further comment and voted collectively on what possible alternative descriptor could be used, recognizing that fundamentally, while certain patterns have great clinical utility, these lesions are heterogeneous and are composed of mixed pathology. Furthermore, their features may be characterized differently by different clinical and research-focused imaging modalities. While it is well appreciated that some will not favor the use of this newly proposed term, all who participated agreed that, given more recent evidence, describing these lesions as DAI is not completely accurate, and therefore there was consensus that an alternative was necessary. The Imaging WG offers this proposal as a first step toward refinement of our radiological terms to describe these lesions; some authors have also proposed the use of this term in pictorial review of TBI imaging CDEs.^13^ Therefore, to promote clinicopathological consistency with current evidence, the working group proposes the combined term TAMVI and urges caution in referring to these lesions identified on CT or conventional MRI alone with terms such as DAI or TAI or traumatic microbleeds.^11,25^ It was appreciated, as has been previously reported, that until advancements in imaging technology can confidently discern the specific pathology, the true extent of axonal injury will likely remain underestimated by imaging modalities such as CT.^13^ The group does believe that this constellation of lesions, in the appropriate distribution and clinical context, helps clinicians to recognize a pattern of injury that has implications for a more accurate diagnosis with practical management and prognostic consequences.

### CDE integration into EMR

The Imaging Working Group proposes that an imaging tool incorporating a well-organized “drop-down menu” of CDE’s^1^ that could be incorporated into radiology-reading software, similar to that available for other imaging contexts (e.g., mammography^27^) would standardize terminology used to describe injuries, link to more detailed information when helpful, and generate standardized radiology reports based on the radiologists’ choices. Given the heterogeneity of brain injury, this menu can flexibly characterize any combination of lesion findings in an individual patient. The tool may also prompt radiologists to report potentially relevant diagnostic and prognostic information. For instance, future renditions could be informed by specific MRI sequences known to detect lesions in an additional 25–30% of patients with unremarkable head CTs at the time of injury and can increase the accuracy of diagnosis and prognostication,^15^ while certain CDEs derived from quantitative sequences (e.g., diffusion tensor imaging-derived measures^28,29^) are likely to provide greater prognostic utility than others. Future updates would incorporate such emerging findings by examining the specificity and utility of newly proposed CDEs (e.g., white matter hyperintensities or enlarged perivascular spaces^4,30,31^), and would allow radiologists to classify whether lesions are preexisting or injury-related, particularly for less severe injury and chronic injury progression.^10^

The proposed tool anticipates that data fields could be added to the general neurotrauma imaging panel to facilitate entry of secondary or more detailed CDE characteristics for more expanded clinical and/or research purposes. Ideally, this initiative would be pursued in parallel with similar applications that could integrate other TBI-related clinical and diagnostic information, such as blood-based biomarkers^32^ given that imaging features are most meaningful when integrated in the broader clinical context. Finally, data fields could be backfilled from radiology reports using tools such as Smart Reporting (https://www.smart-reporting.com/) to increase efficiency, generalizability, and rapid adoption. This working group hopes that this recommendation may be used as motivation to bring together industry partners in a precompetitive space as an important next step to develop this tool for clinical implementation.

### Translation to practice—context of use for clinicians

Incorporating imaging findings into management decisions and prognostication depends on recognizing *patterns of injury* that integrate the wide heterogeneity of imaging findings along with premorbid patient factors, specific injury factors such as mechanism of injury, treatment variability, and newer data such as blood-based biomarkers. An ideal imaging tool would allow clinicians to contextualize the imaging findings to both the temporal phase of injury and to nonimaging factors to characterize the injury more accurately in multiple interlinked dimensions. To this end, the current effort will also incorporate “clinical caveats” into the user-facing neurotrauma imaging tool that will augment interpretation of the implications of specific imaging findings in light of patient factors, such as age or use of particular medications (e.g., anticoagulants) and modifiers such as social determinants of health. In addition, the working group will provide guidance about emerging imaging techniques that might be considered (e.g., advanced MRI techniques in “CT-negative” patients with elevated blood-based biomarkers or persistent symptoms). Imbuing such a tool with relevant and validated ancillary information will require ongoing international collaboration as well as a standardized mechanism for regular updating. The Working Group believes that with the rapid evolution of advanced computational tools such as artificial intelligence (AI), this represents an attainable goal that will enhance radiologist efficiency in using such a tool in practice, optimize clinical care, and provide prognostic counseling for patients with TBI worldwide.

### Communication with patients and families

An important consideration in any new neuroimaging approach is communicating with patients and families/surrogate decision makers. For many, experiencing a TBI causes anxiety, which can be exacerbated by inconsistent nomenclature used by clinicians or encountered in the lay press and media. For example, “concussion” is defined differently between specialties, with varying clinical implications and expected trajectories of recovery.^33^ The word “mild” affixed to “TBI” is equally problematic, as it is associated with a broad spectrum of injury subtypes and widely variable prognoses.^13,34–37^ In studies of physician–patient communication, mismatches have been identified between physicians and patients regarding knowledge and understanding of their diagnoses,^38^ particularly when medical terminology includes words used by the popular press.^39^ Accordingly, the Imaging Working Group recommends future efforts to consider how best to communicate technical pathoanatomic terms to patients and families so as to enhance consistency and comprehension. It is also important to consider that the classification, developed initially in English, will be used for a global health problem, and rates of TBI are highest in low- and middle-income countries (LMICs)^40^ where English is frequently not the population’s first language.

## Recommendations

• Update and reorganize the existing definitions of neuroimaging CDEs to incorporate new findings as well as usability considerations for key time windows postinjury (see Supplementary Appendix).
• Create a neuroimaging tool incorporating the updated CDEs into a drop-down menu format of “basic” findings, with linked standardized definitions (Table 1) integrated into the EMR/PACS (Picture Archiving and Communication System). This tool would be interactive and multilevel, with the basic level (first column; *n* = 18), supplementary level (second column) appearing if the basic lesion was indicated to be present, and emerging level (third column) appearing if the supplementary level was selected so that radiologists can record the location, size, and number of identified lesions.
• Identify software partners and stakeholders who can advise on implementation steps to make the tool accurate, usable, efficient, and helpful to inform the multitude of patient care pathways for patients and providers along the continuum of TBI patient care.
• Devise feature clusters with diagnostic, prognostic, and therapeutic implications linked to clinical, demographic, injury classification, and treatment variables to optimize management decisions and prognostication. Current evidence^41,42^ suggests the following imaging patterns as important feature clusters to consider:
  ◦ Often associated with incomplete recovery in Glasgow Coma Scale (GCS) 13–15: contusion, subdural hemorrhage, and subarachnoid hemorrhage.
  ◦ Often associated with more severe impairment in GCS 13–15: intraventricular hemorrhage and TAMVI.
  ◦ Often associated with incomplete recovery in GCS 12–15: epidural hemorrhage.
  ◦ Often associated with more severe impairment in GCS 3–12: midline shift and basal cistern compression.
• Create a mechanism to “translate” neuroimaging definitions into lay language to bridge communication gaps with patients as well as clinicians from other health care specialties.
• Identified knowledge gap: There was less evidence for key imaging features and how they relate to initial outcome (up to 1-year postinjury) and chronic neurotrauma phases (greater than 1-year postinjury), as compared with the body of evidence supporting associations between imaging features in the acute phase (0–24 h postinjury), which almost exclusively pertains to CT. Further investigation is advised using imaging modalities beyond CT to elucidate the acute neuroimaging features that are most relevant for predicting recovery of function and/or evidence of post-traumatic neurodegeneration in the chronic setting.
• Identified limitations in technology: Specific limitations in current imaging modalities were identified pertaining to spatial resolution and sequence parameters, which should motivate future work to refine our standard acquisition approaches. For example, even the highest resolution clinical imaging at 1 mm in-plane is off by a factor of 1000 from the brain injury tissue pathology that occurs on the order of microns. Another example is reduced-radiation CT scans used in young children, which have generally lower resolution and may tip clinicians toward early MRI in that age group.

**Table 1.** Proposed Template for TBI Neuroimaging Features

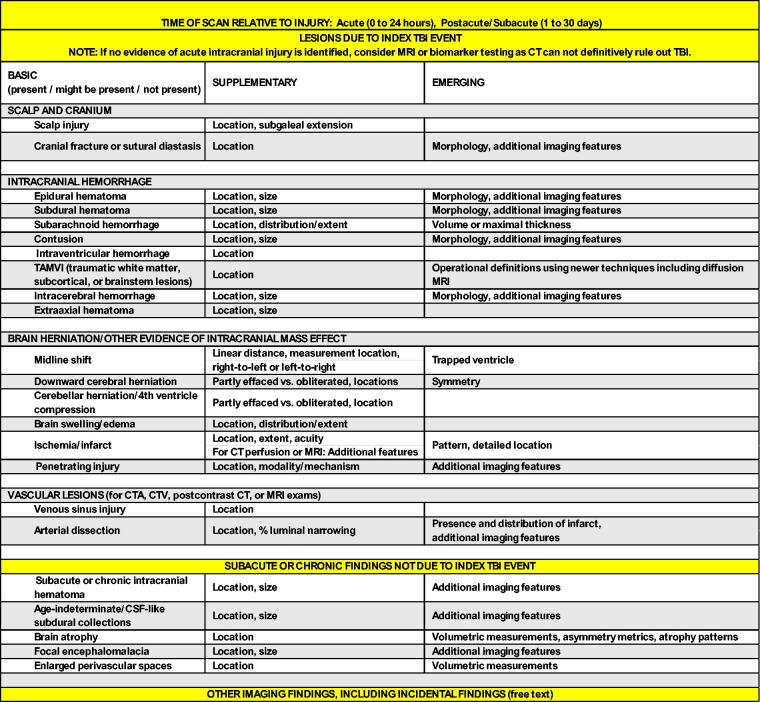		

CT, computed tomography; TAMVI, traumatic axonal and/or microvascular injury; TBI, traumatic brain injury; CSF, cerebral spinal fluid.

## Future Considerations

Besides the goals of clear and consistent terminology and more clinically informative multimodality characterization of TBI, there is an additional need for advances in imaging acquisition and analysis to facilitate the optimal use of brain imaging data in both research and clinical practice. With more advanced imaging sequences such as diffusion MRI (dMRI; inclusive of diffusion tensor imaging and other variants) and resting-state functional MRI, there is a need for harmonization of data collection, analysis, and interpretation of quantitative findings. A particular challenge is that quantitative sequences like dMRI currently require control data collected on each specific machine to enable harmonization. Future development of improved phantoms or utilization of a shared control reference standard may mitigate this issue. Involvement of imaging platform vendors will be key to facilitating sequence development and harmonization, as well as to ensuring consistent use of phantoms and quality assurance protocols. Even with postacquisition harmonization, the use of algorithms like ComBat^43^ may be required. For quantitative imaging to be brought widely into clinical practice, automatic pipelines that robustly identify and account for the presence of lesions are required. The large amount of imaging data available worldwide offers an opportunity to advance knowledge about TBI by leveraging AI technologies. Deep learning models (e.g., convolutional neural networks) have the potential to differentiate lesion types, their spatial distribution, volume, and number, which will facilitate more accurate reporting of lesion burden, inform diagnostic stratification, and support personalized treatment strategies.^44,45^ In clinical practice, the use of AI technologies may be particularly important in resource-constrained systems, which are common in LMICs where the vast majority of TBI occurs and where it may be more difficult to access radiological expertise. To facilitate the combination of large data for developing AI technologies, there is a need for comprehensive and effective frameworks for large-scale, international collaboration^46,47^ and data sharing. In particular, federated learning approaches may facilitate data sharing across countries. Imaging is a rich source of information about a patient’s burden of injury after TBI. Improved use of these data will optimize patient stratification, elucidate the pathophysiology and trajectory of TBI, and enrich clinical trials. Importantly, for individual patients, more accurate injury characterization may help to personalize care, aid selection for treatments, and predict long-term outcomes.

## Transparency, Rigor, and Reproducibility Summary

The Imaging WG conducted a narrative review of the literature. The objective of the working group was to assess the current state of the science as it pertains to radiological classification of TBI and make recommendations based on expert consensus, in conjunction with feedback from patients with lived experiences as well as NIH NINDS leadership, the Steering Committee of the TBI Classification and Nomenclature Initiative, and public comment provided following the meeting. It should be acknowledged that the summary and recommendations reflect the published literature queried and the prior experience of those from the Imaging WG, which may include potential biases pertaining to the imaging technology and imaging features discussed. It is expected as further work is done, there will continue to be advances in the technology and thus the diagnostic recommendations for the evaluation of TBI in best practice.
