# Supplementary material for: Neuroimaging Characterization of Acute Traumatic Brain Injury with Focus on Frontline Clinicians: Recommendations from the 2024 National Institute of Neurological Disorders and Stroke Traumatic Brain Injury Classification and Nomenclature Initiative Imaging Working Group
Source: J Neurotrauma. 2025 Jul 9;42(13-14):1056–64. doi: 10.1089/neu.2025.0079 (PMC12409119; doi:10.1089/neu.2025.0079)
Supplement: Supplementary Appendix [file neu.2025.0079_supplementary_appendix.docx]

This appendix builds upon prior foundational work^1^ to provide clear terms and definitions for neuroimaging common data elements (CDEs) of pathoanatomic entities encountered in patients with traumatic brain injury (TBI). While most of these entities are defined based on radiologic findings, typically CT and MRI, they can also be encountered as surgical or autopsy findings. As some CDEs are only relevant to certain postinjury time frames and not to others, the accompanying table may be useful to define different subsets of CDEs that are applicable in the acute assessment (0-24 hours), primary recovery/subacute (1-30 days), initial outcome (1-12 months), and late effects of neurotrauma (greater than 1 year) after injury. For example, categories for pathophysiologic processes that may occur acutely or in a delayed fashion, as well as those that may have occurred prior to the initial TBI are included.

We propose that users of these definitions will enter acute patient data in their Electronic Medical Record (EMR), at least at the “core” level. This EMR data entry process would include all CDEs that provide prognostic value and/or management utility for acute injuries, including the presence or absence of mass lesions, subarachnoid or intraventricular hemorrhage, brain shift, cisternal compression, and brain edema^2-5^. At medical centers where an EMR is not available, the updated CDEs would still benefit clinical care by providing consistent nomenclature, informing prognostication, and facilitating communication with patients.

We acknowledge that different clinicians, centers, and countries may define these lesions differently. Moreover, as technology advances, the CDEs will likely require ongoing revision. The CDE definitions below are thus meant to be a working and evolving document that provides *practical operational definitions*. The goal is to incorporate these CDE definitions into an automated system that interfaces with the EMR (where available) to generate a radiology report template. This template also will provide the foundation for a standardized data repository for clinical research.

In clinical practice, head CT is typically the initial (acute) imaging modality when a patient presents soon after injury as it is cost-effective, faster, and more widely accessible than MRI, has no absolute contraindications, and is highly sensitive to lesions that require neurosurgery and/or monitoring, including intracranial hematomas, brain herniation, and skull fractures. In specific circumstances (e.g., children, repetitive head injuries as can happen in sports, or when more detail is needed for clinical management), MRI may play an initial role. Clinical decision rules are commonly used to determine the need for imaging, with variations in such rules worldwide. Examples include the New Orleans criteria^6^, Canadian CT head rule ^7^, the National Institute for Health and Care Excellence^8^ guideline for head injury and CT in Head Injury^9^. MRI is playing an increasingly important role in the identification of therapeutically and prognostically important lesions and lesions not detectable using CT.

**General format**

1) The following is a list of pathoanatomic lesions; each patient may have multiple lesions entered into the report/database. For each pathoanatomic lesion, the following index includes the operational definition of the lesion ***for purposes of this database***, including how the lesion may appear on different imaging modalities and what relevant descriptors should be used for its location, distribution, quantification, proximate or remote sequelae or associations, evolution over time, and pathophysiology. The intention is to format these elements in an ***interactive drop-down menu*** so that clinicians and investigators can choose and expand only those entities relevant to that patient and the requirements of the specific clinical report or research study. The accompanying table may also be used to simplify/abridge the list of CDEs to a subset that is most relevant to the pertinent postinjury time point. For patients who have multiple lesions of a single type (for instance, multiple contusions), the interactive database will allow for repeating a specific entity type’s entry so that more than one of the same type of entity can be entered and described.

2) If a pathoanatomic entity is suspected but cannot be diagnosed on a given image with a high degree of confidence, the reader can use the “might be present” option. This may occur because the lesion is too small or there are technical limitations (such as finite resolution) or issues (such as motion artifact).

3) If an entity is NOT present, that item simply can be skipped unless its absence is relevant to a specific clinical concern/diagnosis (e.g., no skull base fracture seen in a patient with a clinical question of rhinorrhea) or if it is required for a specific research study. For this reason, the “Not Present” checkbox is listed in parentheses throughout the index.

5) Data can be entered by levels of complexity and detail. The “Core” tier includes descriptors as to the presence, possible presence, or absence of a particular lesion. “Supplementary” and “Emerging” headings include more detail about the location, extent, and other characteristics of the lesion, and some may require specific radiologic equipment or protocols. It is expected that all entries will include at least the Core data. It is also expected that the “Emerging” category will evolve rapidly to include newer techniques that have not been addressed in this set of definitions.

6) Data can be entered *for each scan* obtained on the patient.

**Date/time of study __/__/____ ____:____ (start time)**

**Suggested format:** Day # / MON / YEAR e.g. Sept 12, 2024 would be 12 / SEP / 2024

**Imaging Modality**

Core: (check one)

Noncontrast CT

MRI without gadolinium

Field strength 1.5 T, 3 T, other

Other (complete supplementary fields)

Supplementary:

CT Manufacturer (dropdown menu); Model; Software version

Postcontrast head CT

CT angiogram head

CT venogram head

CT perfusion

MRI Manufacturer (dropdown menu); Model; Software version

MRI field strength

Sequence name – check all that apply

T1-weighted

Post-contrast T1-weighted

T2-weighted

T2-weighted FLAIR

DWI (diffusion-weighted imaging)

T2*-weighted GRE (gradient echo)

SWI (susceptibility weighted imaging) or Equivalent (e.g. SWAN on GE)

QSM (quantitative susceptibility mapping)

Time-of-flight (non-contrast-enhanced) intracranial MR angiogram

Time-of-flight MR venogram

Contrast-enhanced intracranial MR angiogram

ASL (arterial spin labeling perfusion MRI)

DCE (dynamic contrast enhanced perfusion MRI)

DSC (dynamic susceptibility contrast perfusion MRI)

Task-based functional MRI

Resting-state functional MRI

MR Spectroscopy

Additional Imaging techniques and technical information: (free text)

**Neuroimaging Pathoanatomic Lesion Types**

For each lesion, define and describe as noted; if there is more than one of the same type of lesion, describe each separately.

Exemplar images taken from the NIH-funded TRACK-TBI study with exemplars for Scalp Trauma and Penetrating Injury courtesy of Massachusetts General Hospital teaching files.

**NO EVIDENCE OF ACUTE INTRACRANIAL INJURY OR ACUTE SKULL FRACTURE**

Absence of definitive imaging findings should not in isolation rule out the possibility the patient has experienced a traumatic brain injury, especially in the absence of more advanced imaging techniques or other diagnostic methods (e.g., blood biomarkers). Neuroimaging is best interpreted in conjunction with clinical history, findings and outcomes, blood-based biomarkers, patient reported experience of the index event, and other clinical information.

**ACUTE SCALP TRAUMA**

Definition**:** Any injury to the scalp including lacerations, avulsions, subgaleal hematoma, cephalohematoma or penetration of a foreign body presumably caused by an impact to the head.


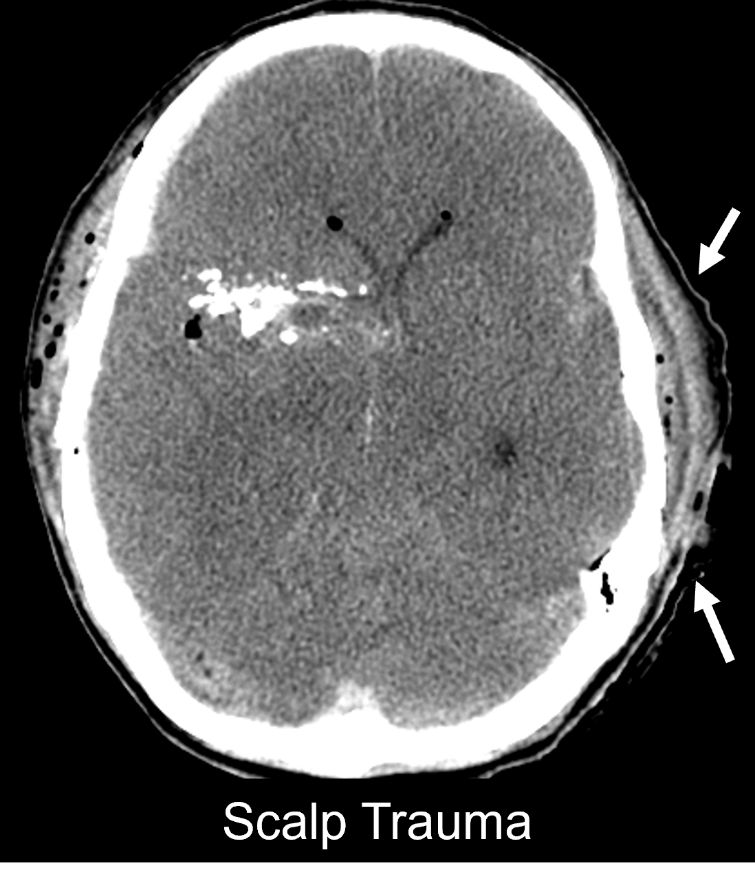


Core:

Is Present.

Might be Present.

Not Present.

Supplementary:

*Location* (check all that apply)

*Frontal R L*

*Parietal R L*

*Temporal R L*

*Occipital R L*

*Subgaleal extension* Yes No

# ACUTE CRANIAL FRACTURE


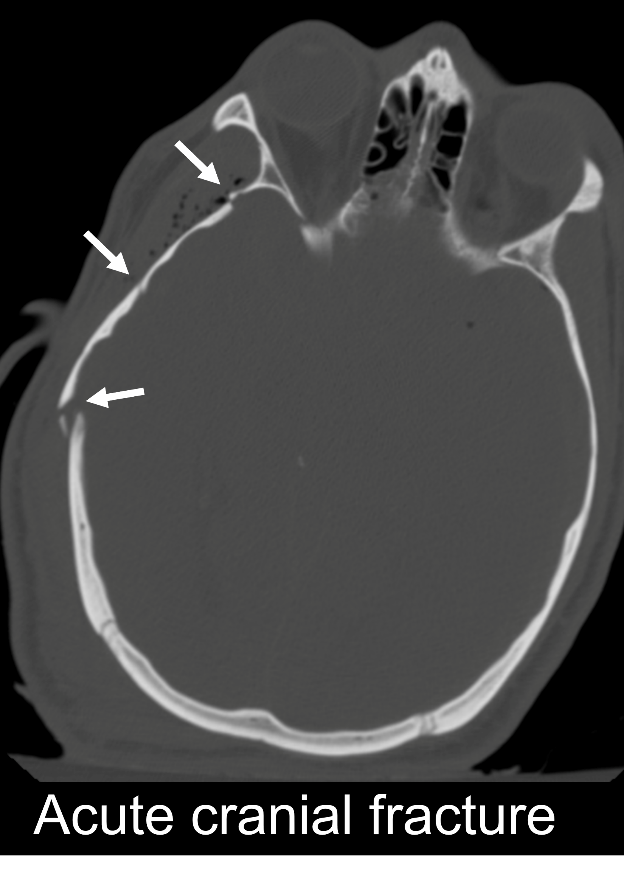
Definition**:** A break in the normal integrity of the calvarium or skull base, presumably caused by mechanical force.

Core:

Is Present.

Might be Present.

Not Present.

Supplementary:

*Location* (check all that apply; for separate fractures, list each separately; for single fractures crossing midline or region, list both sides and/or regions.)

*Skull Base*

*Anterior Fossa R L*

*Middle Fossa R L*

*Posterior Fossa R L*

*Cranial Vault (calvarium)*

*Frontal R L*

*Parietal R L*

*Temporal R L*

*Occipital R L*

Emerging:

*Morphology (check all that apply)*

Linear (includes simple and branched)

Depressed (>1 cm or full thickness of the skull displaced toward the brain)

"Ping pong" or “pond” fracture (smooth depression typically seen in infants and toddlers, without a complete bony cortical disruption)

Comminuted (involving at least one separate non-contiguous bone segment)

Diastatic (encompasses diastatic sutures, with or without adjacent fractures and fractures with widely separated edges. For sutures, consider symmetry with the contralateral side and age of patient)

Compound (communication with the skin, mastoid air cells, or paranasal sinuses)

Penetrating (resulting from an indriven foreign body, such as knife or missile)

“Probable fracture” – one in which fracture itself cannot be seen definitively, but is suspected to be present based on other findings such as adjacent subgaleal and extra-axial hemorrhage, intracranial air, or other findings

Pneumocephalus

Present

Absent

#

# ACUTE INTRACRANIAL HEMORRHAGE

## Epidural Hematoma (EDH) (also known as extradural hematoma)


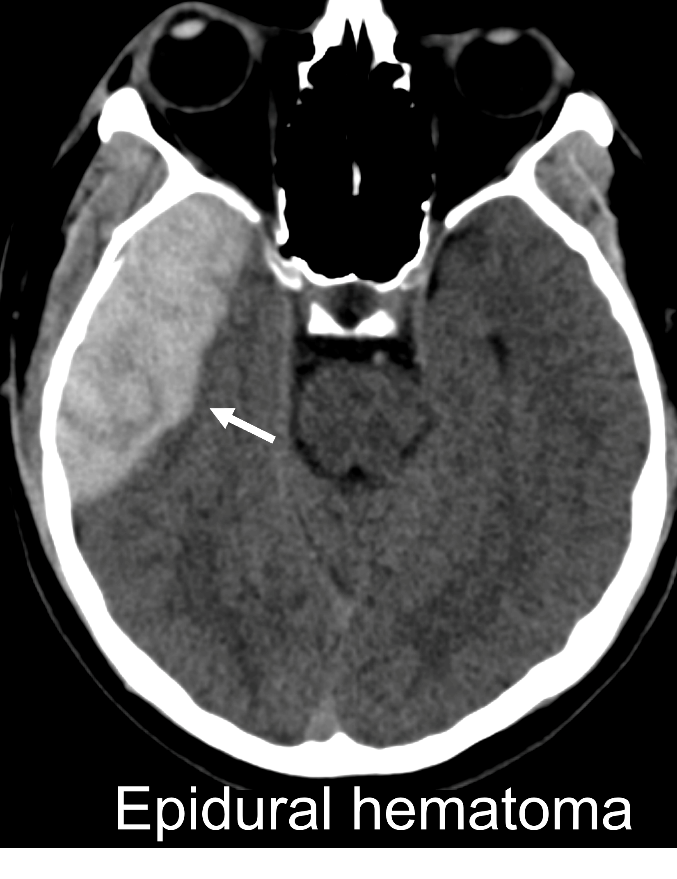
Definition: A collection of blood between the skull and dura. It typically does not cross sutures, though this rule may not apply in the case of comminuted or displaced skull fractures, or in children with certain fracture patterns.

EDH typically (though not always) has a biconvex shape and an overlying skull fracture. Acute EDH is typically hyperdense on CT but may contain hypodense areas representing uncoagulated blood. As the EDH ages, it gradually loses its CT hyperdensity. Although its internal signal characteristics on MRI vary, the dura can be visualized immediately subjacent to the EDH as a thin line that is hypointense on all MRI pulse sequences.

Core:

Is Present

Might be Present.

Not Present.

Supplementary:

*Location* (check all that apply; for separate lesions, list as separate entries):

Frontal R L

Parietal R L

Temporal R L

Occipital R L

Posterior fossa R L

*Size*

Volume (or length, width, and maximal thickness)

Emerging:

Likely arterial (due to “swirl”, different densities, location near major dural artery)

Likely venous (due to association with adjacent bony injury/fracture, venous sinus, size, distribution, timing)

## Subdural Hematoma (SDH)


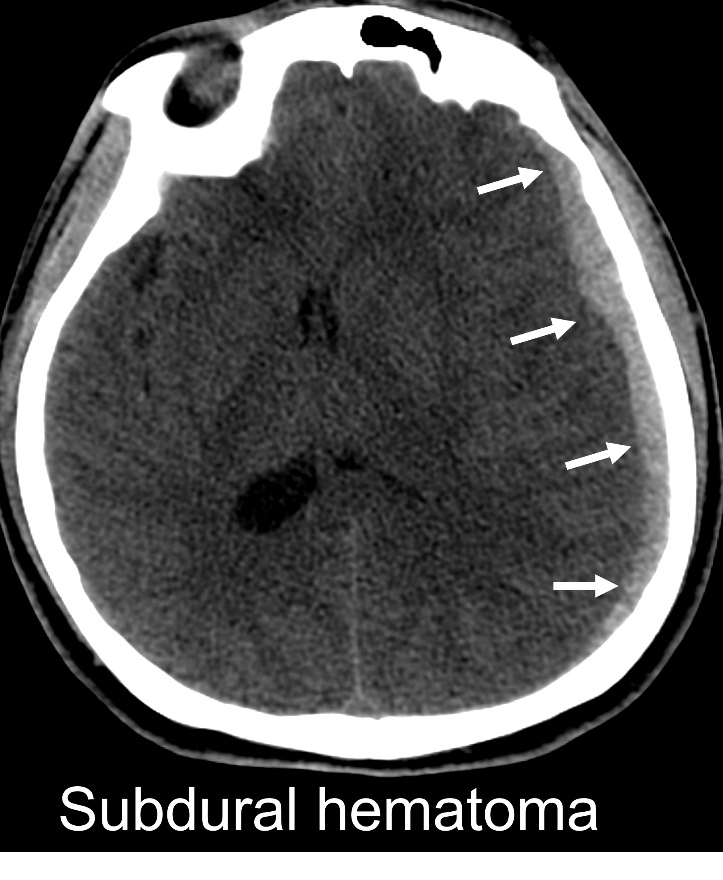
Definition: A collection of blood between the arachnoid and the dura, typically (though not always). On CT, acute SDH is hyperdense and, when large, is often crescent-shaped. Mixed density may be seen if the collection contains unclotted blood, CSF admixture, active extravasation, and/or subacute or chronic components. MRI signal characteristics are variable.

Note: Please see additional categories below for subacute, chronic, and mixed collections if these better describe the lesion, or if the chronicity/timing is uncertain.

Core:

Is Present

Might be Present.

Not Present.

Supplementary:

*Location* (check all that apply; for separate lesions, including separate chronicity, list as separate entries so separate recording for acute and prior injury SDH):

Frontal R L

Parietal R L

Temporal R L

Occipital R L

Interhemispheric supratentorial

Anterior (frontoparietal) Posterior (occipital)

Tentorial R L

Posterior fossa R L Interhemispheric Infratentorial

*Size* Volume (or length, width, and maximal thickness).

Note: When limited to a single measurement, consistently prioritize measuring the maximal thickness, as this may be used as a determinant for assessing the need for surgical evacuation.

Emerging: Homogeneous or Heterogeneous

## Extraaxial Hematoma


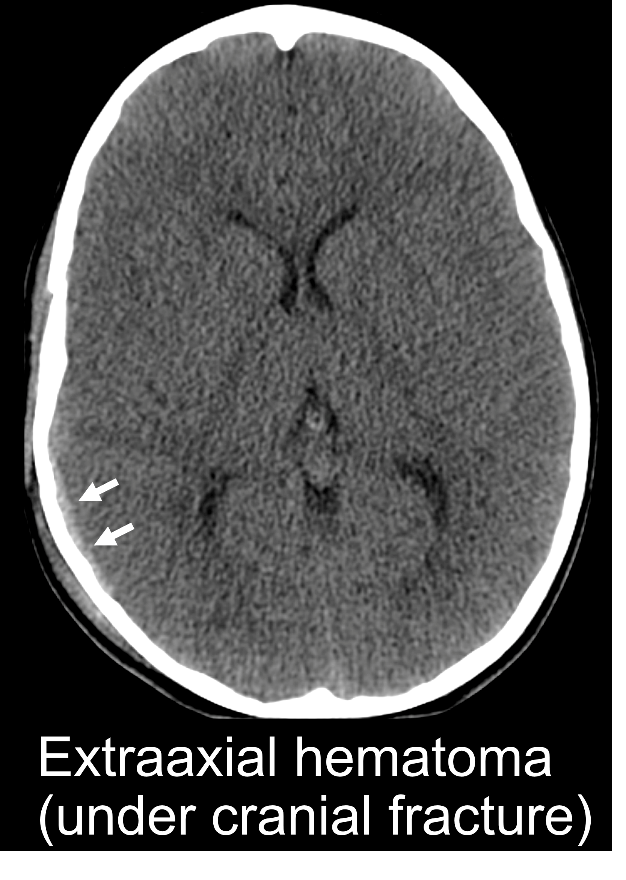
Definition: Sometimes the exact site of collection of blood cannot be determined with certainty. A collection of blood between the brain surface and the skull which may be subarachnoid, subdural, or epidural, and cannot be classified as a more specific entity. These are typically small in volume.

Core:

Is Present.

Might be Present.

Not Present.

Most likely true pathoanatomic type:

Subarachnoid hemorrhage

Subdural hemorrhage

Epidural hemorrhage

Supplementary:

*Location* (check all that apply; for separate lesions, list as separate entries):

Frontal R L

Parietal R L

Temporal R L

Occipital R L

Interhemispheric supratentorial

Anterior (frontoparietal) Posterior (occipital)

Tentorial R L

Posterior fossa R L Interhemispheric Infratentorial

*Size*


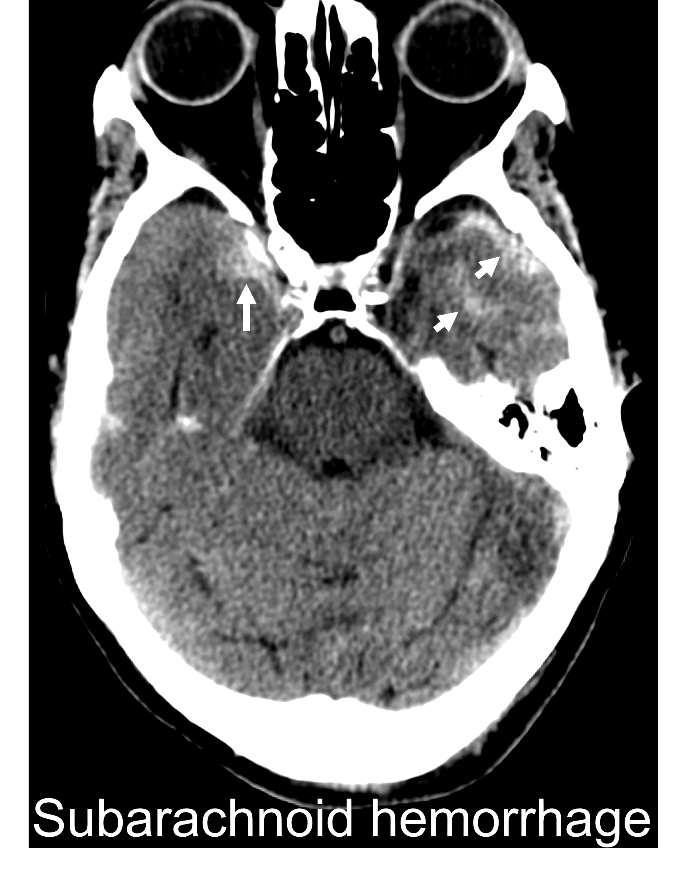
Volume (or length, width, maximal thickness)

## Subarachnoid Hemorrhage (SAH)

Definition: Macroscopic blood located between the brain surface and the arachnoid membrane. SAH often follows the contour of the sulci and cisterns. Acute SAH is hyperdense on CT. Subacute SAH may be invisible on CT, although the presence of subtle sulcal “effacement” may occasionally be seen. On MRI, acute SAH is hyperintense on T2-weighted FLAIR. In the chronic stage, SAH may result in or “hemosiderosis,” which may be appear as curvilinear hypointense areas of cortical “staining” on GRE and SWI.

Core:

Is Present.

Might be Present.

Not Present.

Supplementary:

*Location* (check all that apply):

Frontal R L

Parietal R L

Temporal R L

Occipital R L

Interhemispheric Anterior (frontoparietal) Posterior (occipital)

Tentorial R L

Sylvian fissure

Suprasellar

Perimesencephalic

Prepontine

Other posterior fossa

*Distribution/extent*

Focal (in 1-2 locations or lobes of the brain)

Diffuse (involving *more than two* contiguous lobes or brain regions, supra- and infratentorial compartments, or multiple basal cisterns)

Emerging:

Total volume

(maximal) thickness

## Intraventricular Hemorrhage


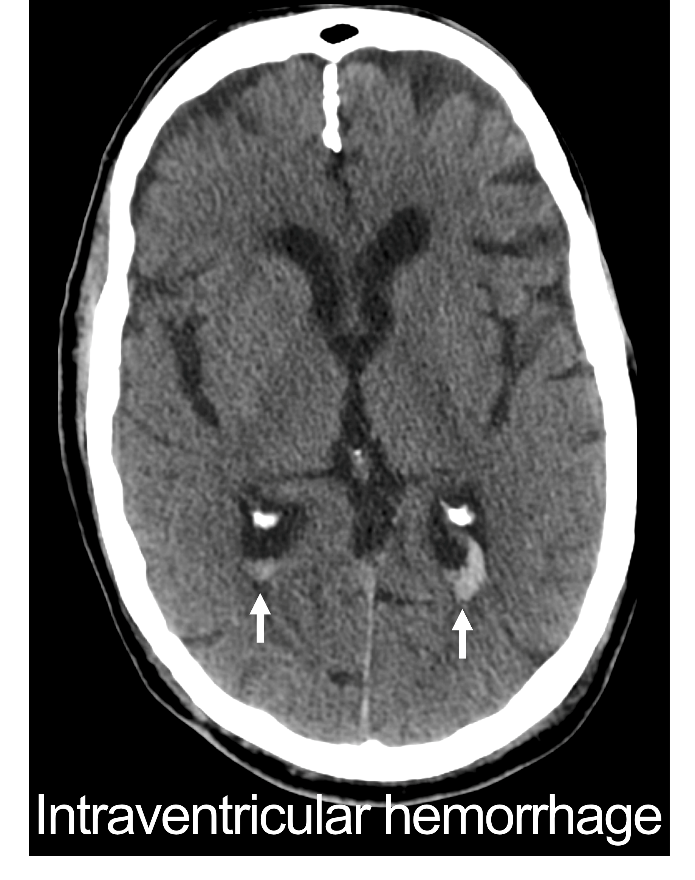
Definition: Hemorrhage within the ventricular system. On CT, acute IVH is typically hyperdense, often in dependent parts of the ventricular system (e.g., occipital horns, atria) or along the septum pellucidum. IVH is often associated with traumatic microbleeds (as defined below). IVH often appears hyperintense on T2-weighted FLAIR and hypointense on susceptibility-weighted MRI sequences but depends on time since injury.

Core:

Is Present.

Might be Present.

Not Present.

Supplementary: *Location* (check all that apply):

Lateral ventricle R L

Third ventricle

Fourth ventricle

Along septum pellucidum

Emerging:

Not enough evidence to support a recommendation.

## Contusion


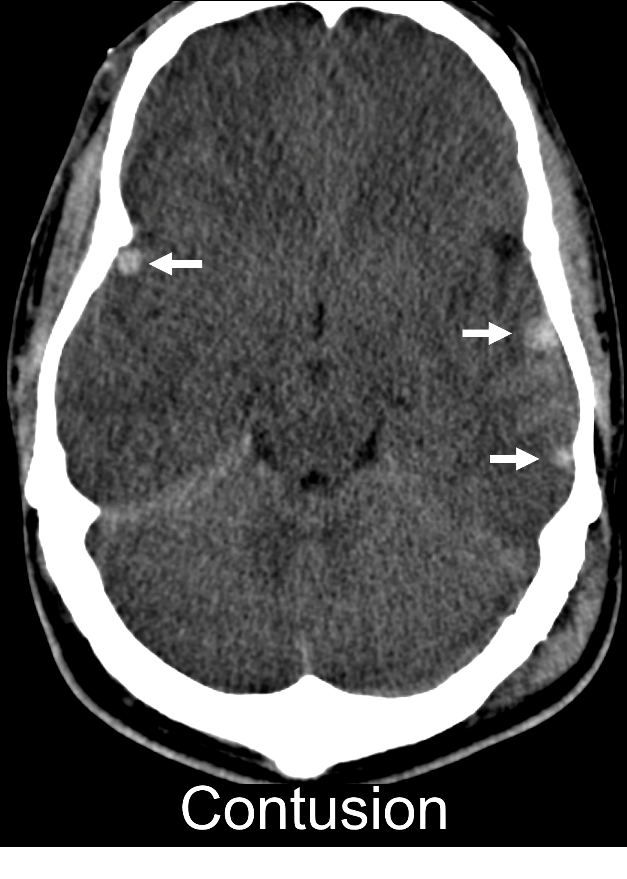
Definition: A focal area of brain parenchymal disruption due to acute mechanical deformation. Contusions typically occur in the cerebral cortex and may extend into subcortical or deeper regions. Acute contusions typically have a mottled, inhomogeneous appearance due to stippling of blood along the brain surface. As such, their size is difficult to measure. The term “contusion” should not be used for hemorrhagic lesions which fit better in other categories, such as small or large hemorrhages associated with the pattern of traumatic microbleeds, or non-traumatic intracranial hemorrhage. Contusions, however, are commonly seen with other lesions such as adjacent SAH and depressed skull fractures. For purposes of categorization, contusions are also differentiated from “intracerebral (or intraparenchymal) hematoma” by containing a mixture of hemorrhage and non-hemorrhagic brain tissue, unlike intraparenchymal hematoma which will refer to non-traumatic brain hemorrhages (e.g., hemorrhage due to vascular malformation or cerebral amyloid angiopathy; and hematomas that are predominantly uniform collections of blood perhaps most commonly associated with hypertensive or drug-induced vasculopathy, or occasionally in venous infarction due to cerebral vein or sinus thrombosis). Ischemic infarcts with hemorrhagic transformation are classified below under hypoxic/ischemic injury.

Appearance on CT: Acutely, contusions are initially hyperdense or heterogeneous, patchy and/or bearing ill-defined margins, often enlarging (sometimes dramatically, known as “blooming”) and/or developing more well-defined borders, usually within the first 6 to 12 hours postinjury. Small contusions that are not visible on the initial CT may become apparent on follow-up CT. Contusions which are questionable, such as those in an area of beam hardening on CT scan, should be noted as “indeterminate.” After approximately 24 hours, hemorrhages typically begin to develop surrounding hypodensity that represents vasogenic edema and/or aging blood products along the periphery of the hematoma. Contusions in which the hemorrhagic component enlarges over time should not be reclassified on subsequent images as "intraparenchymal hemorrhage."

Appearance on MRI: Small cortical contusions may only be visible on MRI, particularly on T2- or susceptibility-weighted sequences acutely; and on these in addition to T1-weighted sequences in the subacute stage.

Core:

Is Present.

Might be Present.

Not Present.

Supplementary:

*Location* (check all that apply; for separate lesions, list as separate entries):

Frontal R L

Parietal R L

Temporal R L

Occipital R L

Cerebellum R L

Brainstem R L

*Size*

Volume (or length, width, maximal thickness) (Note: measurements should include *all* areas of contiguous abnormality not related to a separate lesion, including surrounding edema)

Emerging: (Check all that apply)

Hemorrhagic

Non-hemorrhagic

Cortical

Subcortical

Deep brain structures

Brainstem

Probable brain laceration (linear hemorrhagic or non-hemorrhagic pattern, often associated with overlying skull fracture)

## Traumatic Axonal and/or Microvascular injury (TAMVI) (superficial and deep white matter, subcortical structures, and brainstem)

Definition: We propose the term TAMVI to denote focal lesions in the superficial and deep white matter, subcortical structures, and brainstem, most often associated with inertial forces that cause high magnitude angular acceleration/deceleration of the head and shearing injury in the brain.

The terminology of these lesions (previously referred to variously as DAI or TAI, petechial hemorrhages, punctate hemorrhage, shear injury, traumatic vascular injury, traumatic microbleeds, and microhemorrhages) has evolved over time. New findings, particularly from radiological-pathological correlation studies, show that when associated with hemorrhage, these focal lesions are in fact a mix of pathologies when interrogated at the microscopic tissue level, with some representing purely microvascular injury, some demonstrating both microvascular injury and axonal injury, and others congested vessels.


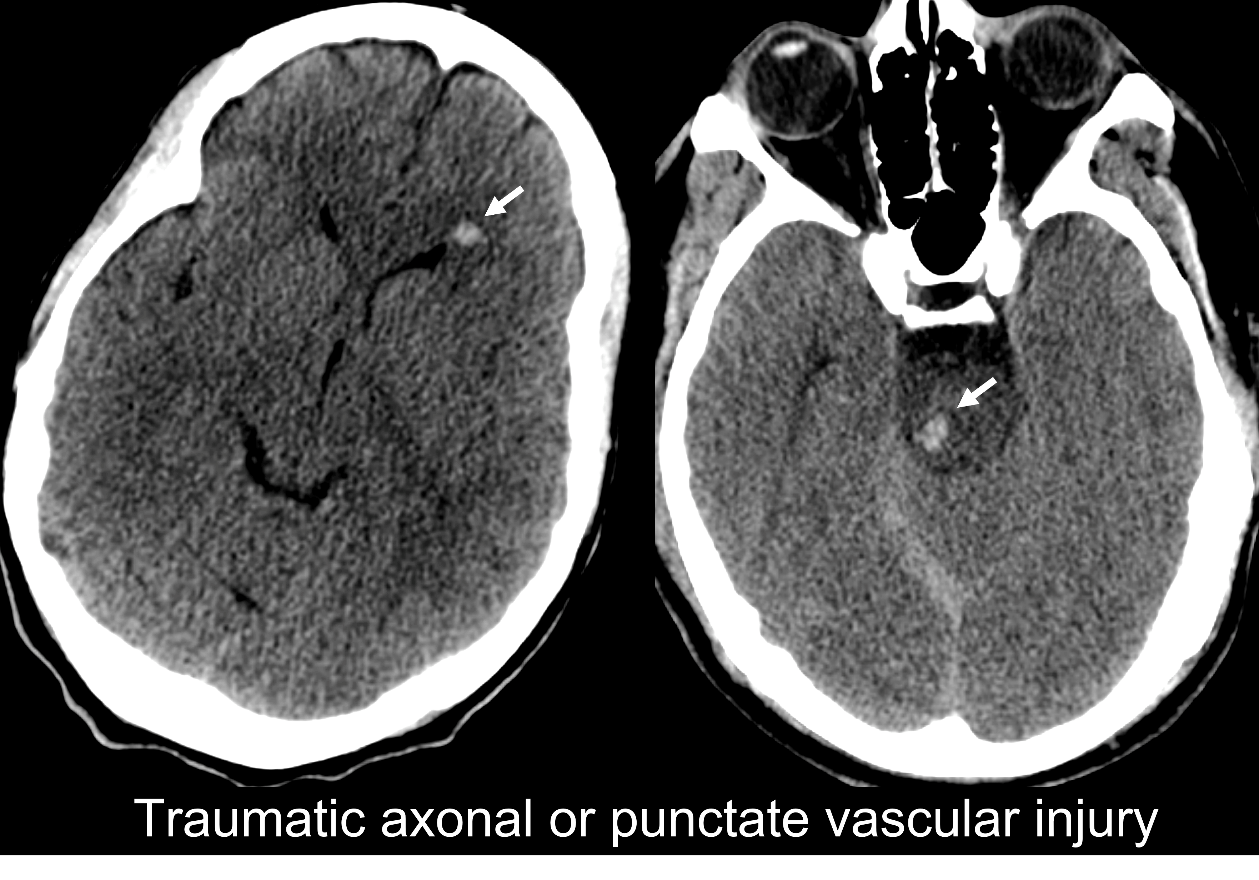


Traumatic Axonal and/or Microvascular Injury


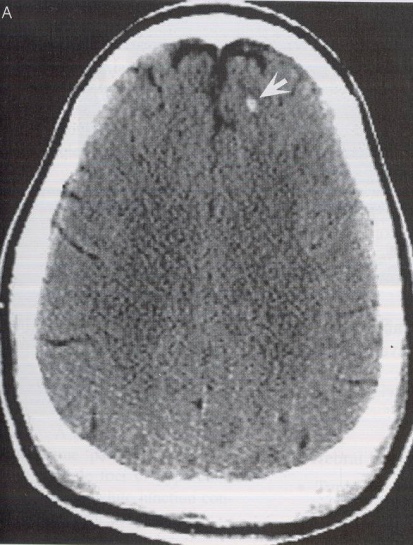


Alternately, focal lesions with the same distribution of typical locations but without associated hemorrhage, generally invisible on CT, may manifest solely as T2-weighted or T2-weighted FLAIR hyperintense lesions. Within 24 to 48 hours postinjury, these lesions often also demonstrate reduced diffusion (i.e., low intensity on apparent diffusion coefficient (ADC) and high intensity on diffusion-weighted imaging (DWI). There is certainly overlap in appearance of these T2-hyperintense lesions with other non-traumatic pathological entities, so another necessary element is a distribution that does not fit better with other common pathological processes, such as small-vessel infarcts and chronic small vessel ischemic disease in older patients.

In previous CDE definitions, TAI was used to describe 1 to 3 lesions, while DAI was used to refer to ≥4 lesions not limited to only one region of the brain, whether or not the lesions were associated with hemorrhage.

Although much remains to be understood regarding these lesions that have been found to demonstrate heterogeneous histopathology and likely different pathophysiology, we recommend the term TAMVI to refer to most cases previously referred to as “TAI,” “DAI,” traumatic microbleeds, petechial hemorrhages, etc., for several reasons: 1) separate terms for lesions without and with blood products is awkward and impractical for radiologists and other readers, as these lesions frequently co-occur in the same patients (and may arise from substantially similar mechanical forces on the brain); 2) they share many imaging features including T2 hyperintensity, reduced diffusion on early MRI at 24-48 hours, and the same characteristic locations; 3) the prior cutoff of ≥4 lesions, while reasonable, is somewhat arbitrary, and better understanding of the strength of the correlation between number/location of these focal lesions on CT and structural MRI and histopathological evidence of “diffuse” white matter injury is needed, and 4) as discussed above, small focal hemorrhages may or may not demonstrate associated axonal injury histopathologically.

While terms such as DAI will likely persist, we urge caution,**^10,11^** as the underlying pathophysiology is heterogenous and outcomes are highly variable, even in patients with many or even widespread lesions. When superficially located, ≥4 lesions can be associated with minimal neurological deficits.

Appearance on CT: Hyperdense foci or curvilinear lesions, typically located in the subcortical white matter (most often frontal, followed by parietal and temporal), and corpus callosum, and sometimes in the basal ganglia, thalami, fornix, and brainstem.

Appearance on MRI: On MRI, they may appear as foci or curvilinear areas of susceptibility artifact, best detected with T2*-weighted GRE, SWI, SWAN, or QSM. They are typically 5-10 mm in diameter or smaller and often, but not always, have surrounding T2-weighted or T2-weighted FLAIR hyperintensity (best visualized with T2-weighted FLAIR).

Alternately, focal T2-hyperintense lesions may not demonstrate susceptibility artifact on MRI, but may manifest solely as T2-weighted or T2-weighted FLAIR hyperintense lesions (or, within 24-48 hours of injury, reduced diffusion (i.e., low intensity on apparent diffusion coefficient (ADC) and high intensity on diffusion-weighted imaging (DWI)) in the characteristic locations listed above. There is certainly overlap in appearance of these traumatic lesions with other non-traumatic pathological entities, so another necessary element is a distribution that does not fit with other common pathological processes in older individuals, such as small-vessel infarcts and chronic small vessel ischemic disease.

Related terms: Also previously referred to as DAI/TAI, petechial hemorrhages, punctate hemorrhage, shear injury, traumatic vascular injury, traumatic microbleeds, and microhemorrhages. The term “gliding contusion,” formerly used to refer to small traumatic microbleeds in the subcortical white matter attributed to angular rotation, is discouraged due to potential confusion with “contusion,” a separate and distinct pathoanatomic lesion.

Core:

Is Present.

Might be Present.

Not Present.

Supplementary:

Location: (mark signal abnormalities identified by each imaging sequence in each location: e.g., DWI, CT, T2-weighted FLAIR, T2*-weighted GRE, SWI, SWAN, QSM, T1-weighted-Gd)

| Imaging Sequence (e.g. CT, SWI, …): | Definite | | Possible | |
| --- | --- | --- | --- | --- |
|  | Right | Left | Right | Left |
| **Subcortical Lobar** |  |  |  |  |
| Frontal |  |  |  |  |
| Parietal |  |  |  |  |
| Temporal |  |  |  |  |
| Occipital |  |  |  |  |
| **Deep White Matter** |  |  |  |  |
| Corpus Callosum: Genu |  |  |  |  |
| Corpus Callosum: Body |  |  |  |  |
| Corpus Callosum: Splenium |  |  |  |  |
| Fornix |  |  |  |  |
| Internal Capsule: Anterior Limb |  |  |  |  |
| Internal Capsule: Posterior Limb |  |  |  |  |
| **Basal Ganglia** |  |  |  |  |
| Caudate Nucleus |  |  |  |  |
| Putamen |  |  |  |  |
| Globus Pallidus |  |  |  |  |
| Thalamus |  |  |  |  |
| **Brainstem** |  |  |  |  |
| Mesencephalon |  |  |  |  |
| Pons |  |  |  |  |
| Medulla |  |  |  |  |
| Other: |  |  |  |  |
| **Cerebellum** |  |  |  |  |
| Cerebellar Peduncles |  |  |  |  |
| Central White Matter |  |  |  |  |

Emerging:

*Overall assessment:*

Operational definitions of TAMVI involving newer techniques including advanced diffusion MRI acquisitions.

## Intracerebral Hemorrhage


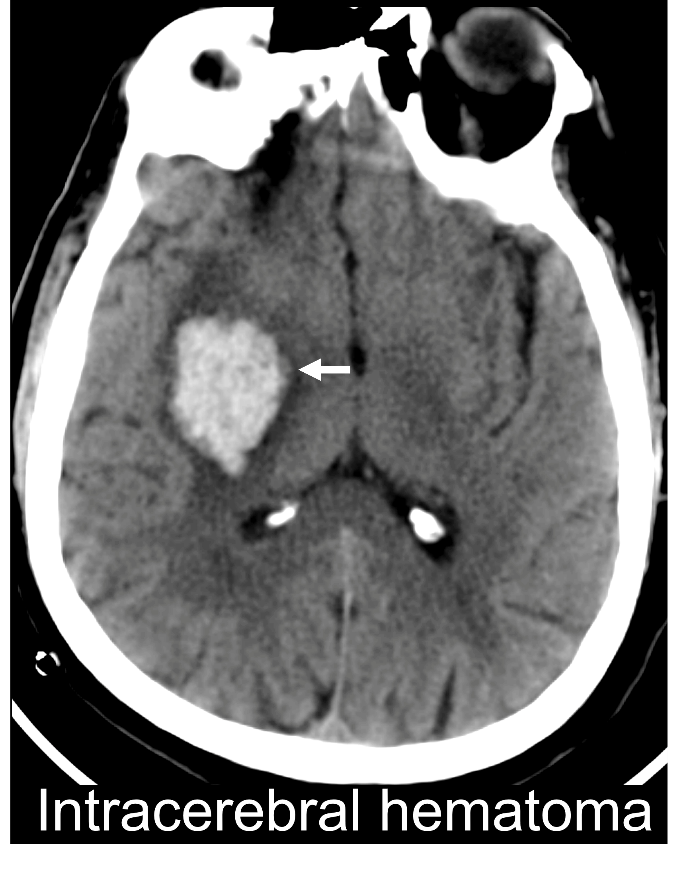
Definition: A collection of confluent, relatively homogeneous blood within the brain parenchyma. Intracerebral hemorrhage can occur in the setting of brain laceration along with other types of brain injury, and there is some overlap with other entities. In general, lesions characterized by mixed blood and tissue are generally classified as contusions. In most instances, the term “intracerebral hemorrhage” is used to refer to larger collections of blood (typically, more than about 10 mm). Hemorrhages can have a surrounding region of non-hemorrhagic signal abnormality that may represent edema or clot retraction. Very small collections more often occur in the setting of contusion or, when scattered throughout the brain, may represent diffuse injuries, often associated with high magnitude rotational forces or other strain/shear forces, that can affect blood vessels and/or axons (see TAMVI section.

Core:

Is Present.

Might be Present.

Not Present.

Supplementary:

*Location* (check all that apply; for separate lesions, list as separate entries):

Frontal R L

Parietal R L

Temporal R L

Occipital R L

Internal capsule R L

Thalamus R L

Basal ganglia R L

Midbrain R L

Pons R L

Medulla R L

Cerebellum R L

*Size*

Volume (or length, width, maximal thickness) of hemorrhagic component

Volume (or length, width, max thickness) of entire lesion, including surrounding signal abnormalities.

Emerging: (Check all that apply)

Layered (i.e., with fluid level)

Surrounding ring of non-hemorrhagic signal (edema)

# ACUTE BRAIN HERNIATION OR OTHER INTRACRANIAL MASS EFFECT


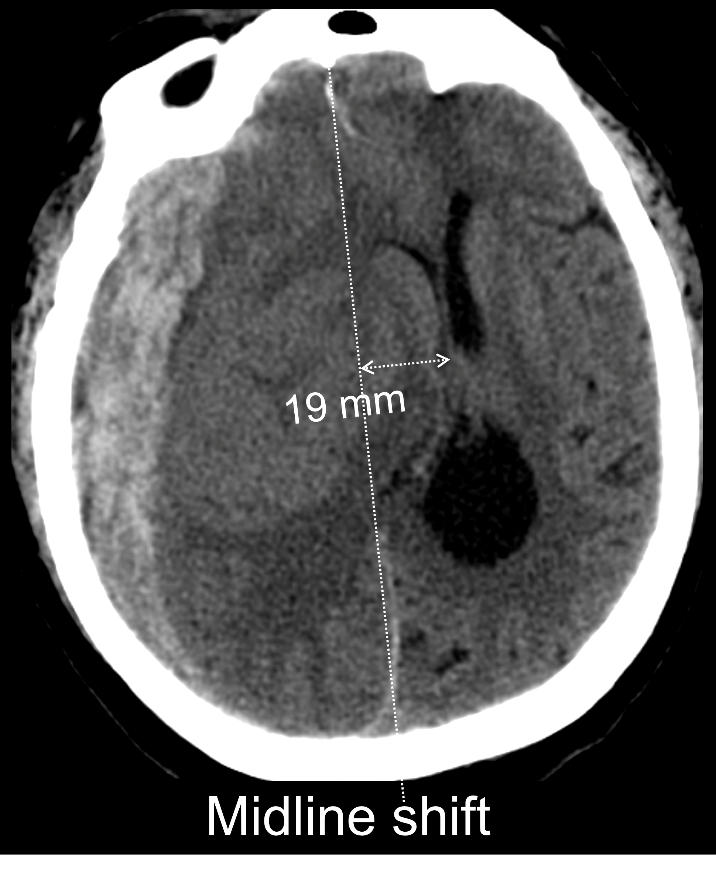
Definition: Brain herniation refers to any displacement of cerebral and/or cerebellar tissue from its normal anatomical location into an adjacent space due to increased intracranial pressure, mass effect and/or brain swelling. Major types of brain herniation include: 1) midline shift, 2) downward cerebral and 3) upward or downward cerebellar herniation. External, transcalvarial herniation may also occur, when brain tissue extends outside the expected contour of the cranial vault due to open skull fracture or craniectomy. “Midline shift” and “subfalcine” herniation are often used interchangeably, though the latter specifically refers to displacement of the cingulate gyrus under the free edge of the falx cerebri. Downward cerebral herniation refers to downward displacement of the central or lateral parts of the cerebrum due to mass effect from a focal traumatic lesion and/or brain swelling or edema. Increased pressure/mass effect in the posterior fossa can be manifested as upward displacement of the cerebellum into the supratentorial space via the tentorial incisura (“ascending transtentorial” herniation) and/or downward displacement of the tonsils through the foramen magnum (“downward tonsillar” herniation”). When seen in the acute post-injury phase or in the context of a large mass lesion, this can suggest the need for urgent intervention, while in more chronic situations shifts can be better tolerated and require clinical judgment for timing of intervention.

## Midline Shift

Definition: Displacement of the supratentorial midline structures, particularly the septum pellucidum, *3 mm or more* due to mass effect attributable to a focal traumatic lesion or brain swelling/edema. Subfalcine herniation may be present. Shift is measured at the Foramen of Monro, or alternatively, where it is greatest.

Core:

Is Present.

Might be Present.

Not Present.

Supplementary:

*Linear displacement*: ___mm (Foramen of Monro or greatest displacement)

*Side:*

Right-to-left

Left-to-right

*Measured at:*

Septum pellucidum

Pineal gland

Emerging:

When lateral herniation is seen, enlargement of the contralateral lateral ventricle may indicate concomitant ventricular outflow obstruction. Compression of the contralateral brainstem from lateral tissue shift may result in a “Kernohan’s Notch” phenomenon, with hemiparesis ipsilateral to the mass lesion.

## Downward Cerebral Herniation


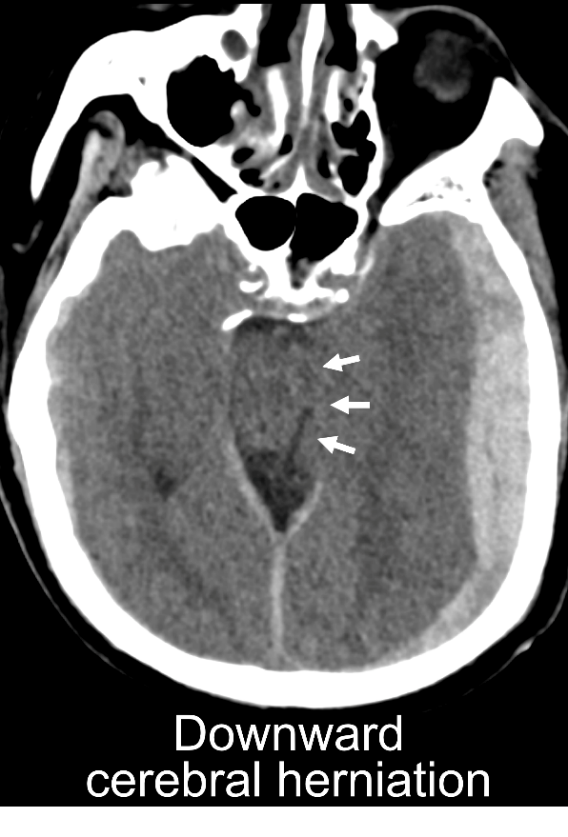
Definition: Downward cerebral herniation refers to downward displacement of the central or lateral parts of the cerebrum due to mass effect from a focal traumatic lesion and/or brain swelling or edema. Partial or complete effacement of the suprasellar, prepontine, perimesencephalic, or superior cerebellar/quadrigeminal cistern due to abnormal descent of part(s) of the cerebral hemisphere due to mass effect in the supratentorial space. The basal cisterns are essential anatomical landmarks, and house clinically important cranial nerves and basal cerebral arteries. Recognition of asymmetry, symmetric effacement, or obliteration of these cisterns is essential to the identification of downward cerebral herniation. Cistern volume relative to overall brain volume should be assessed, as the cisterns enlarge in parallel with age-related brain volume loss. So-called “Duret hemorrhages” may also be seen in the brainstem in this condition.

Core:

Is Present.

Might be Present.

Not Present.

Supplementary:

*Cisternal Compression Severity:*

Partly effaced

Obliterated

Emerging: (Enter site and symmetry for each abnormal cistern separately):

*Site*

Suprasellar

Perimesencephalic/ambient

Quadrigeminal/superior cerebellar

*Asymmetry:*

Left

Right

Left > Right

Right > Left

Right = Left

## Cerebellar Herniation/Compression of 4th Ventricle (posterior fossa mass effect)


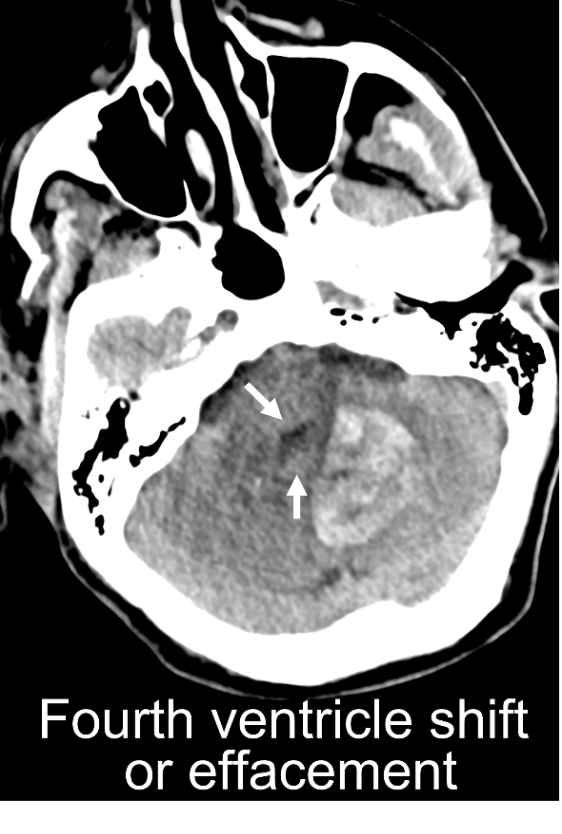
Definition: Increased pressure/mass effect in the posterior fossa can be manifested as upward displacement of the cerebellum into the supratentorial space via the tentorial incisura (“ascending transtentorial” herniation) and/or downward displacement of the tonsils through the foramen magnum (“downward tonsillar” herniation”).

Core:

Is Present.

Might be Present.

Not Present.

Supplementary (Enter site and symmetry for each abnormal cistern and/or ventricle separately):

*Severity:*

Partly effaced

Obliterated

*Site*

Upward transtentorial

Downward tonsillar

Compression/shift of 4th ventricle

___mm (maximal distance from expected location in any direction)

Right-to-left, left-to-right, anterior, posterior

## Brain Swelling/Acute Edema


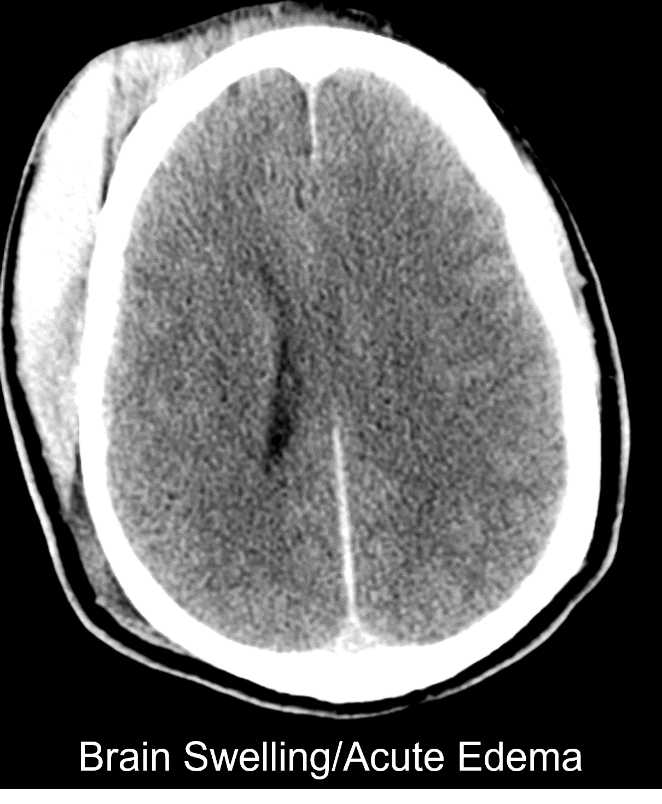


Definition: Acute brain swelling is an all-inclusive term that refers to a non-specific increase in brain tissue mass. It can result from increased water as in the various types of acute cerebral “edema”, but it can also result from “hyperemia” (i.e., increased intravascular blood volume). The latter situation is typically found in venous hypertension in which the tissue is engorged due to outflow obstruction. Cerebral hyperemia can also be found in the dysautoregulated brain when the systemic blood pressure is elevated, and in some hypermetabolic states in which the tissue is hyperperfused. Radiologically, cerebral hyperemia appears as focal or diffuse mass effect (i.e. sulcal/cisternal effacement) with preservation of the gray-white differentiation (GWD). Cerebral edema also appears as focal or diffuse mass effect, but the increased water results in obscuration of the GWD.

This appears as loss of sulci, compression of basal cisterns and flattening of the ventricular margins, but gray/white attenuation and differentiation remain intact. It may result in brain herniation. For cytotoxic edema, it appears hypodense with loss of gray-white matter differentiation.

Core:

Is Present.

Might be Present.

Not Present.

Supplementary: *Location (check all that apply):*

Frontal

R L

Parietal

R L

Temporal

R L

Occipital

R L

Deep gray matter

R L

Cerebellum

R L

Brainstem

*Extent* :

Focal (involves less than half of one lobe)

Lobar (involves more than half of one lobe)

Multilobar (involves multiple lobes)

Hemispheric (involves an entire hemisphere)

Bihemispheric (involves both hemispheres)

Posterior fossa (involves the cerebellum and/or brainstem)


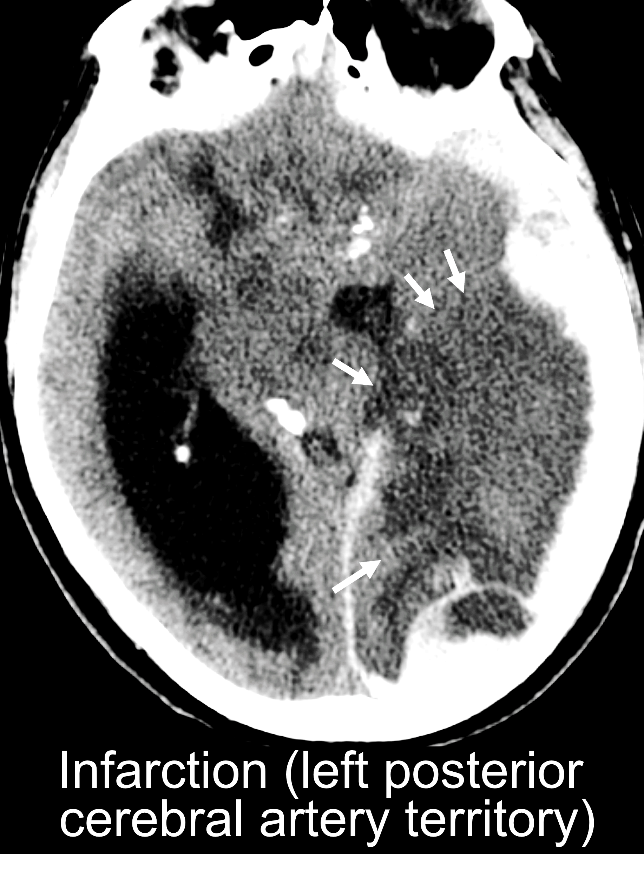
 Global (involves the entire brain)

# OTHER ACUTE LESIONS

## Ischemia/Infarction

Acute trauma also may be associated with hypoxia and/or ischemia from a variety of specific causes, including apnea, herniation, and embolic phenomenon.  For more information see NIH Common Data Elements for Parenchymal Imaging, (i.e. Stroke CDEs)^12^.

Definition: Ischemia and other related terms above refer to findings in tissue which sustains, for a variety of reasons, a deficit between substrate demand and delivery. This may be reversible or irreversible. Examples of specific etiologies include arterial occlusion, embolic infarction, lacunar infarction, watershed infarction, venous infarction, and changes from global insults such as hypoxia, hypotension, intracranial hypertension, status epilepticus, and others. Unlike the bland contusion, the location of the lesion respects a specific vascular territory, and this can be a helpful radiologic clue. The *lacunar* infarct results from occlusion of one of the penetrating arteries or arterioles that provide blood to the brain's deep structures. They are typically less than 1.5 cm in size, ovoid or round in shape, and located in the basal ganglia. The *watershed* infarct results from an episode of systemic hypoperfusion. The lesion is located at the junction of the ACA/MCA/PCA border zones. *Venous* infarction results from reduced outflow of blood from the brain in the setting of cortical and/or dural sinus thrombosis or occlusion. Ischemia from intracranial hypertension may occur when intracranial pressure exceeds mean arterial pressure. This can occur in the setting of a global increase in intracranial pressure, or a focal, compartmental increase (e.g., in the setting of a mass lesion).

Appearance on CT: On CT, the acute *embolic* infarct is seen as an area of hypodensity which becomes more defined with time. On CT, early venous hypertension is typically seen as a subcortical area of hyperemic swelling which may progress to vasogenic edema. Overt venous infarction is often hemorrhagic and multifocal. Therefore, the smaller hemorrhagic venous infarct can mimic traumatic microbleeds. The radiologic manifestation of ischemia from intracranial hypertension is typically loss of grey-white matter differentiation and sulcal effacement on CT, and high intensity on DWI. Other modalities for detection of ischemic injuries include CT and perfusion MRI (e.g., ASL, DCE, or DSC).

Appearance on MRI: MRI features will include changes on various sequences. Petechial hemorrhage and/or overt hemorrhagic transformation may occur, and this will be best seen on T2*-weighted GRE and SWI. On DWI, the acute embolic, watershed, and lacunar infarcts are seen as a focal “light-bulb” bright area. Typically, during the first week the apparent diffusion coefficient (ADC) will also be low. The lesion intensity will fade over time on DWI (typically gone by two weeks) but it will persist on T2-weighted and FLAIR images, and ADC values will also start to increase (Note: DWI may normalize sooner in neonates after ischemia.) The radiologic manifestation of ischemia from intracranial hypertension is typically high intensity on DWI.

Additional Considerations: A “cord sign” or “empty delta sign” may be seen on contrast CT/MR, and CTV and MRV can often reveal the intraluminal thrombus, hypodensities in arterial distributions, or those in the pattern of venous thrombosis. Other modalities for detection of ischemic injuries include CT and perfusion MRI (e.g., ASL, DCE, or DSC).

Core:

Is Present.

Might be Present.

Not Present.

Supplementary:

*Location (check all that apply):*

Frontal

R L

Parietal

R L

Temporal

R L

Occipital

R L

Deep gray matter

R L

Cerebellum

R L

Brainstem

*Extent*:

Focal (involves less than half of one lobe)

Lobar (involves more than half of one lobe)

Multilobar (involves multiple lobes)

Hemispheric (involves an entire supratentorial hemisphere)

Bihemispheric (involves both hemispheres)

Posterior fossa (involves the cerebellum and/or brainstem)

Global (involves the entire brain)

*Acute vs. subacute*

*For CT: (check all that apply)
 Hypodense*

*Isodense*

*Hyperdense*

*Mixed*

For CT perfusion:

Decreased CBF and reduced CBV

*For MRI: (check all that apply)*

*T1-weighted hyperintense isointense hypointense mixed*

*T2-weighted hyperintense isointense hypointense mixed*

*FLAIR hyperintense isointense hyperintense mixed*

*DWI hyperintense normal mixed*

*ADC hyperintense hypointense*

Emerging:

*Pattern:*

Watershed

Arterial

Lacunar

Venous

Dissection

Mixed

Indeterminate

Detailed location by gyral anatomy template

##

##
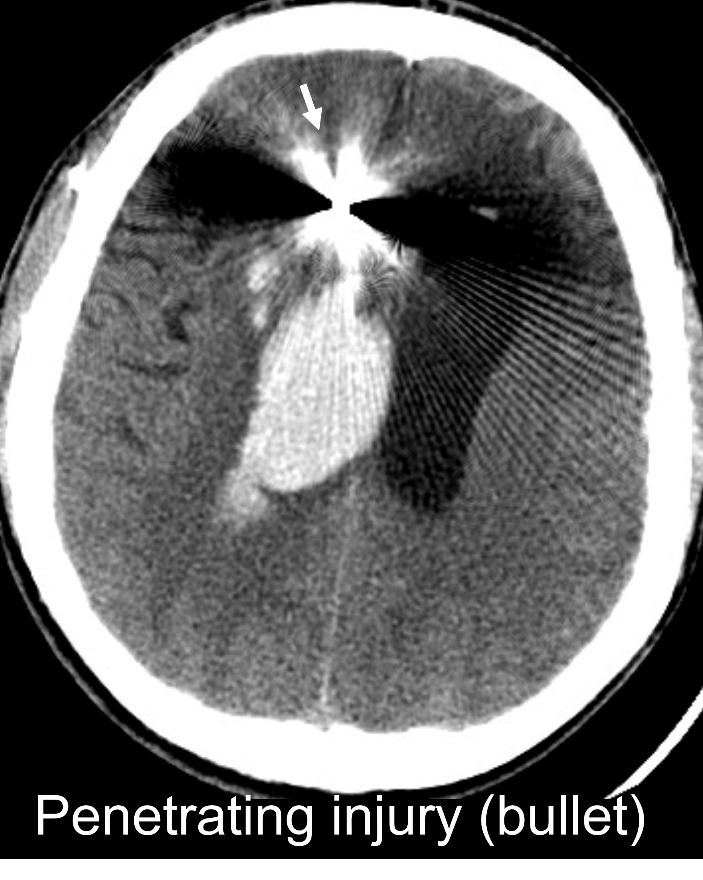
Penetrating Injuries

Definition: Injuries caused by traumatic forces which penetrate any of the normal layers of the head, including skull, dura, and brain. Examples include gunshot wounds, other missiles and projectiles, stab wounds, and other penetrating objects.

Core:

Is Present.

Might be Present.

Not Present.

Supplementary:

*Location* (check all that apply):

Frontal R L

Parietal R L

Temporal R L

Occipital R L

Internal capsule R L

Thalamus/Basal ganglia R L

Midbrain R L

Cerebellum R L

Pons R L

Medulla R L

*Modality/mechanism*

Stab wound

Gunshot wound

Caliber/type___

Other foreign body____

Emerging:

Indriven fragments (bone, foreign bodies)

Through and through trajectory (entrance and exit sites)

Transventricular trajectory

Crosses midline

# VASCULAR LESIONS

Generally applicable only if CT/MR/catheter angiogram study or postcontrast CT is being evaluated.

## Arterial Dissection


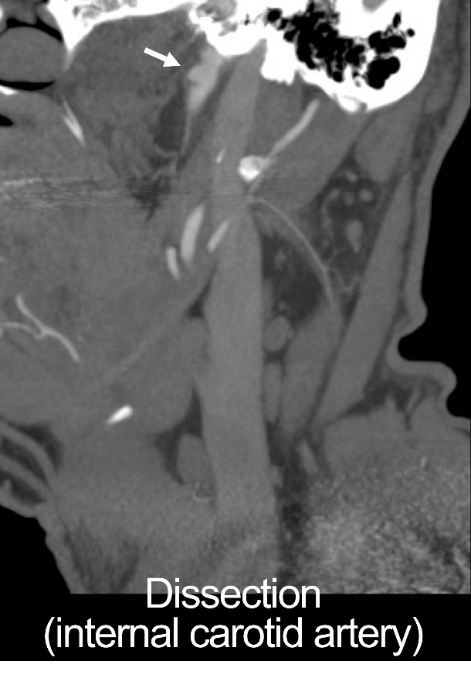
Definition: Disruption of one or more inner layers of an artery, which may be traumatic or spontaneous. CTA, MRI, and MR angiography (MRA) may show an abnormally small or irregular caliber of the injured artery, pseudoaneurym, occlusion, or transection. A “crescent sign” may be seen on axial MRI (and less well with CTA) and is best identified on T1-weighted Fat-Saturation images. If the caliber of the lumen is unaffected, catheter angiography may miss the vascular dissection, and the diagnosis may be visualized only with CTA/MRI.

Biffl et al.^13^ is the most commonly used grading system for these lesions:

Grade I: Luminal irregularity or dissection resulting in <25% luminal narrowing

Grade II: Dissection resulting in ≥25% luminal narrowing, intraluminal thrombus, or raised intimal flap

Grade III: Pseudoaneurysm

Grade IV: Arterial occlusion

Grade V: Arterial transection

**
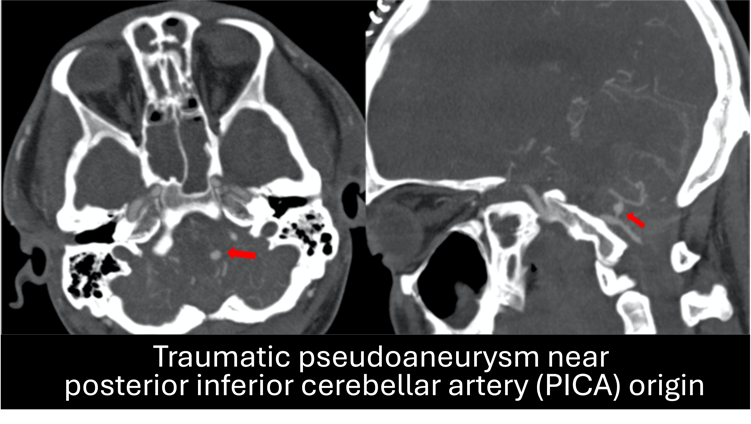
**Traumatic pseudoaneurysm (Biffl Grade III) is a focal outpouching of an artery due to mechanical disruption of all layers of the arterial wall with extravasation of blood into a confined soft-tissue space. CTA, MRI/MRA, postcontrast CT, and catheter angiography reveal a focal collection of contrast adjacent to the vessel. In contrast to non-traumatic aneurysms, the dilated wall of a pseudoaneurysm may have an irregular surface, and the lesion is not located in typical berry aneurysm locations. Intraluminal thrombus of varying ages can appear as laminated rings of varying signal intensity on MRI. Phase artifact, indicative of pulsation within the lesion, may be seen on MRI. Peripheral wall calcification may be seen in older pseudoaneurysms and is best visualized with CT or, in some cases, conventional angiography. (Figure, slightly revised, from Palmieri M, Pesce A, Zancana G et al., Neurosurgical Review 2022;45:1019-1029 under Creative Commons 4.0 International License.)

Note: If more than one vessel has dissection, list each separately.

Core:

Is Present.

Might be Present.

Not Present.

Supplementary:

*Location* (check all that apply; for separate lesions, list as separate entries):

Carotid R L

Vertebral R L

ACA R L

MCA R L

PCA R L

Basilar

Other (Describe) R L

*Biffl grade*

Luminal narrowing < 25% (Biffl Grade 1)

Luminal narrowing > 25% (Biffl Grade 2)

Pseudoaneurysm (Biffl Grade 3)

Arterial occlusion (Biffl Grade 4)

Arterial transection (Biffl Grade 5)

*Associated findings*

Watershed or embolic infarction in the territory of the dissected vessel

+/- SAH

Adjacent skull fracture (e.g., carotid canal)

Adjacent vertebral fracture (e.g., vertebral foramen)

Size (mm, length of involved vessel)

Intraluminal thrombus

Cavernous (intradural)

## Venous Sinus Injury

Definition: Disruption of any one of the venous sinus vessels which drain blood from the cranial cavity thought to occur from abutting skull fractures but also reported in closed head injury. On CT it can appear subtle and is often found by irregularity in the hyperdense signal of the venous cavity. CTV, MRI/MRV are also used to visualize this injury where filling defects can be observed.


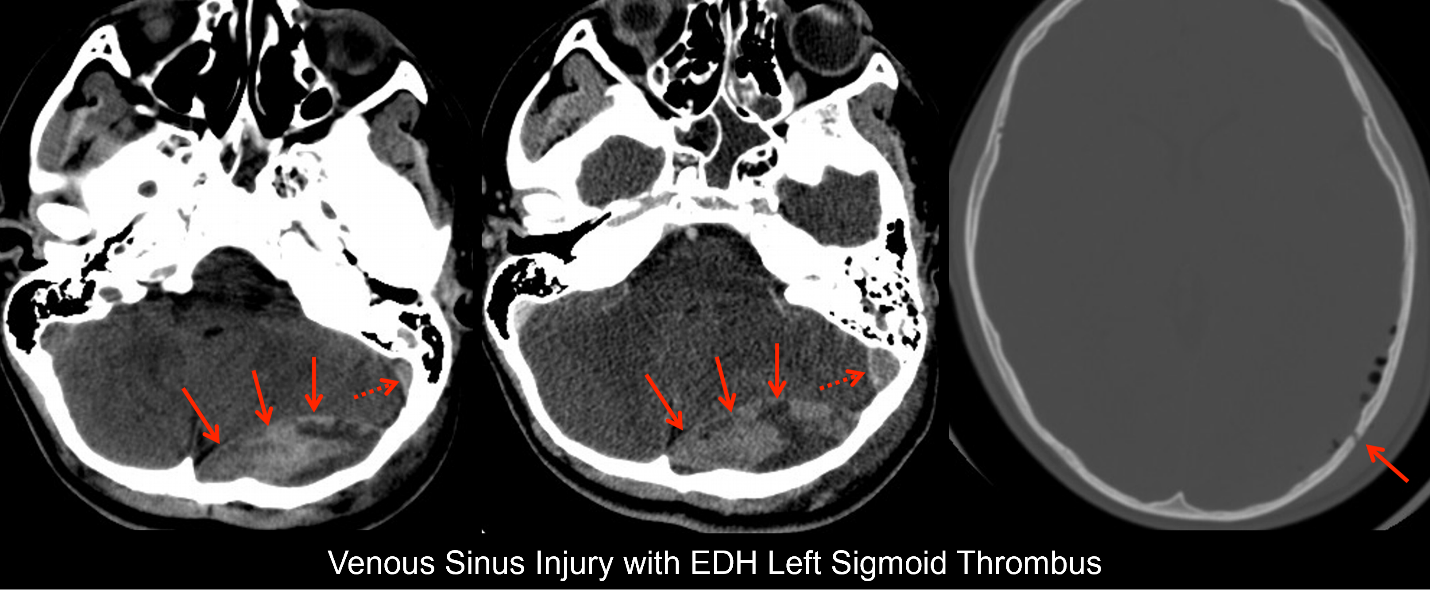


Core:

Is Present.

Might be Present.

Not Present.

Supplementary:

*Location* (check all that apply;

Superior Sagittal

Inferior Sagittal

Straight

Transverse

Sigmoid

Cavernous

Superior Petrosal

# Imaging Findings Potentially Arising from Non-Acute TBI or other Prior Injuries

It is common upon review of Head CT or Brain MRI for a possible acute brain injury that there may also be imaging signatures of prior injuries as well. This section is included to address the ‘mixed chronicity’ that a clinician can encounter in reviewing brain imaging.

## Non-Acute Intracranial Hematoma (Subacute, Chronic)


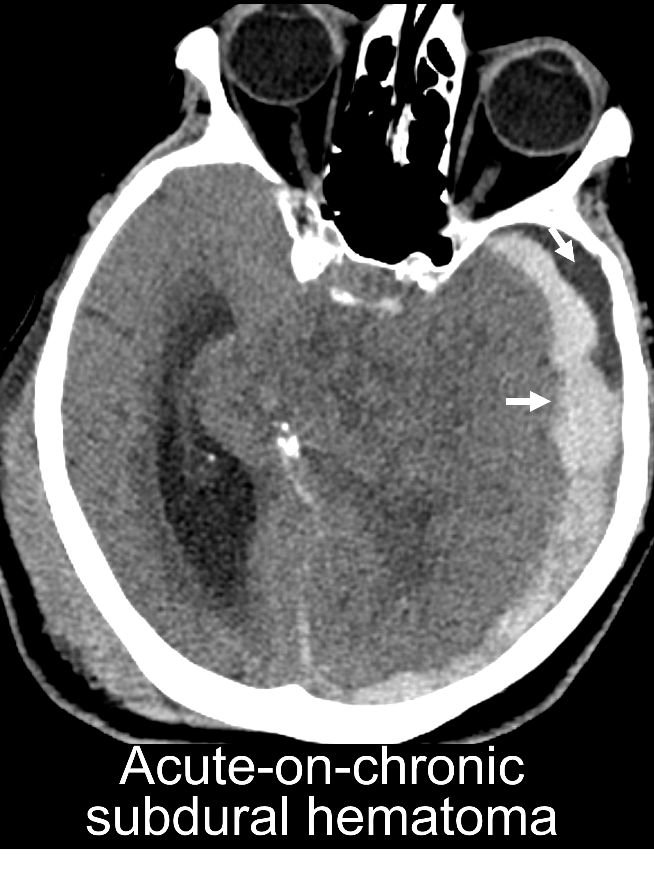
Definition: A collection of non-acute blood between the arachnoid and the dura, typically (though not always) with a crescent shape.

Appearance on CT: On CT, a subacute or chronic SDH will be predominantly iso- or hypodense.

Appearance on MRI: On MRI, a subacute SDH will be hyperintense on T1-weighted and will have varying signal intensity on T2-weighted imaging. The chronic SDH is slightly hyperintense compared to CSF on both T1- and T2-weighted imaging. T2-weighted FLAIR imaging increases conspicuity. If rebleeding has occurred in the collection (i.e., “chronic recurrent SDH”), the signal may be a variable combination of hypo/iso/hyper-density/intensity on CT and MR sequences, respectively.

Internal loculations and septations may be seen on both CT and MRI, and these are more conspicuous following intravenous contrast enhancement.

Note: This definition does NOT apply to CSF-intensity collections or prominent spaces seen on a single image, which may represent entities other than trauma. (See also the section on Atrophic Changes below.)

Core:

Is Present.

Might be Present.

Not Present.

Supplementary:

*Location* (check all that apply: for separate lesions, list as separate entries):

Frontal R L

Parietal R L

Temporal R L

Occipital R L

Interhemispheric

Anterior (frontoparietal) Posterior (occipital)

Tentorial R L

Posterior fossa R L Interhemispheric Infratentorial

*Size*

Volume (or length, width, and maximal thickness)

Note: When limited to a single measurement, consistently prioritize measuring the maximal thickness.

Emerging:

Homogeneous v. Heterogeneous

If heterogeneous:

Hypointense/dense

Hyperintense/dense

Isointense/dense

Loculations/Septations

## Age-indeterminate/CSF-like subdural collections

##


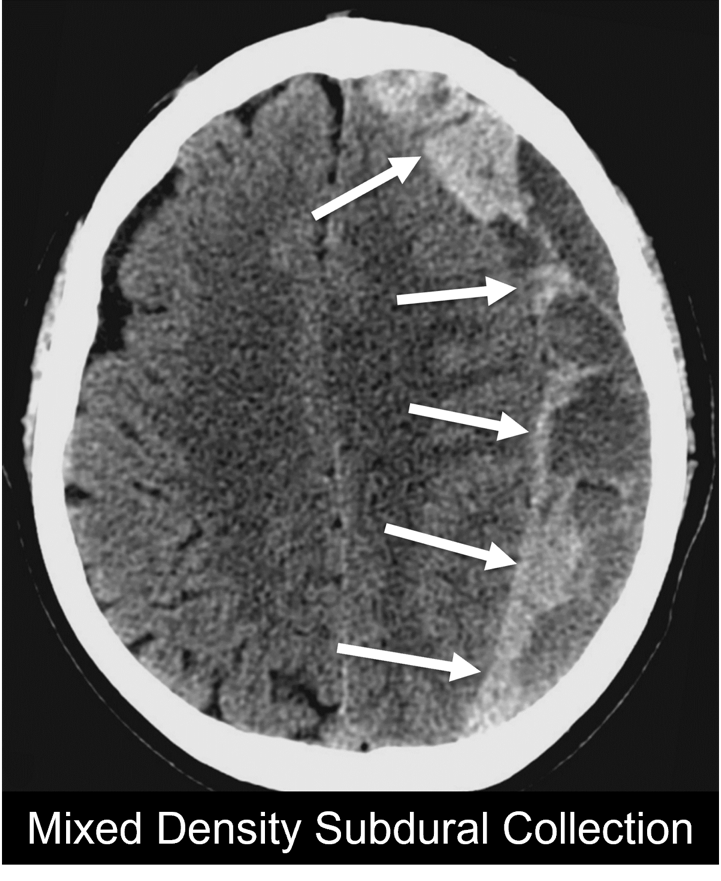
Definition: A collection of inhomogeneous or CSF-like fluid between the arachnoid and the dura, typically (though not always) with a crescent shape, in which timing (e.g., “acute” vs. “chronic” or “subacute”) is indeterminate. On CT and MRI, mixed collections may have hyper, iso, or hypodense/intense components. This classification is used for those collections in which the exact nature of the collection or its chronicity cannot be determined by the characteristics noted in the definitions of subdural hematomas in the two prior sections. In addition to mixed collections, more homogeneous CSF-density/intensity collections also may be seen *after known acute trauma* in which low density/intensity collections occur over time on serial images, presumably from arachnoid tears, decreased CSF absorption, increased CSF protein, or other mechanisms. This definition does NOT apply to CSF-intensity collections or prominent spaces seen on a single image, which may represent entities other than trauma. (See also section on Brain Atrophy below.)

Core:

Is Present.

Might be Present.

Not Present.

Supplementary:

*Location* (check all that apply; for separate lesions, list as separate entries):

Frontal R L

Parietal R L

Temporal R L

Occipital R L

Interhemispheric Anterior (frontoparietal) Posterior (occip)

Tentorial R L

Posterior fossa R L

*Size*

Volume (or length, width, maximal thickness)

Emerging:

Characteristics (check all that apply)

Hypointense/dense

Hyperintense/dense

Isointense/dense

## Focal Encephalomalacia


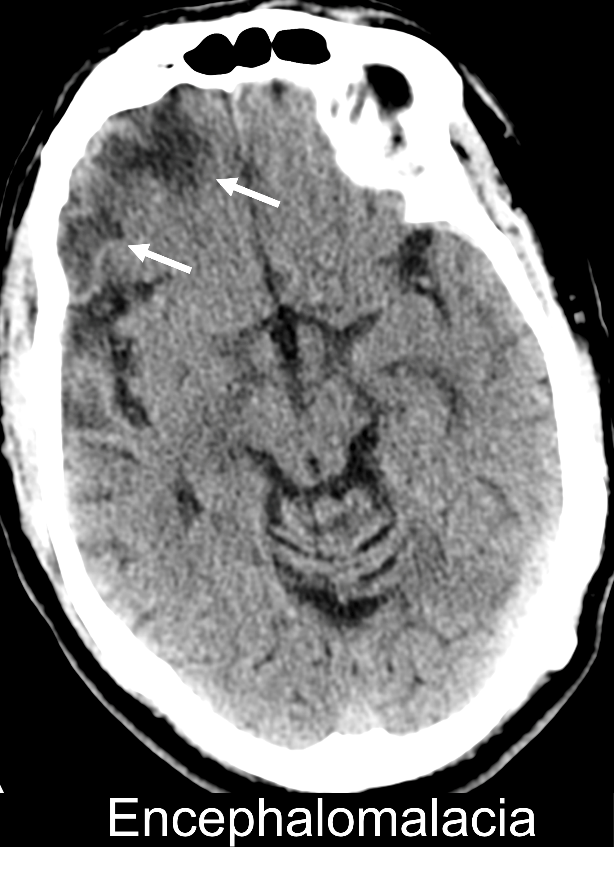
Definition: A term coined by pathologists that refers to loss of brain tissue after cerebral infarction, cerebral ischemia, infection, traumatic brain injury or other injuries. Typically, the affected parenchymal tissue undergoes liquefactive necrosis, resulting in a clearly defined lesion that consists of fluid, necrotic tissue or pus.

Appearance on CT: On CT, the affected parenchymal tissue will appear hypodense with a slightly higher attenuation than CSF. There is typically noticeable volume loss, which may present with or without Wallerian degeneration and/or gliosis. Wallerian degeneration is evident as atrophy of the ipsilateral cerebral peduncle, while gliosis is seen as an area of somewhat reduced attenuation on CT.

Appearance on MRI: On MRI, the affected tissue that is considered lost follows CSF signal on all sequences including FLAIR. Wallerian degeneration is visible as high intensity on DWI in the acute phase, and gliosis appears as hyperintense on T2-weighted imaging and FLAIR, with low signal on T1-weighted imaging and facilitated diffusion on ADC. Gliosis manifests as atrophic tissue displaying a high T2 signal.

Core:

Is Present.

Might be Present.

Not Present.

Supplementary: *Location (check all that apply):*

Frontal R L

Parietal R L

Temporal R L

Occipital R L

Cerebellum R L

Cerebral peduncle R L

Volume or linear measurements

Emerging:

Multicystic

Wallerian degeneration

Gliosis

## Brain Atrophy


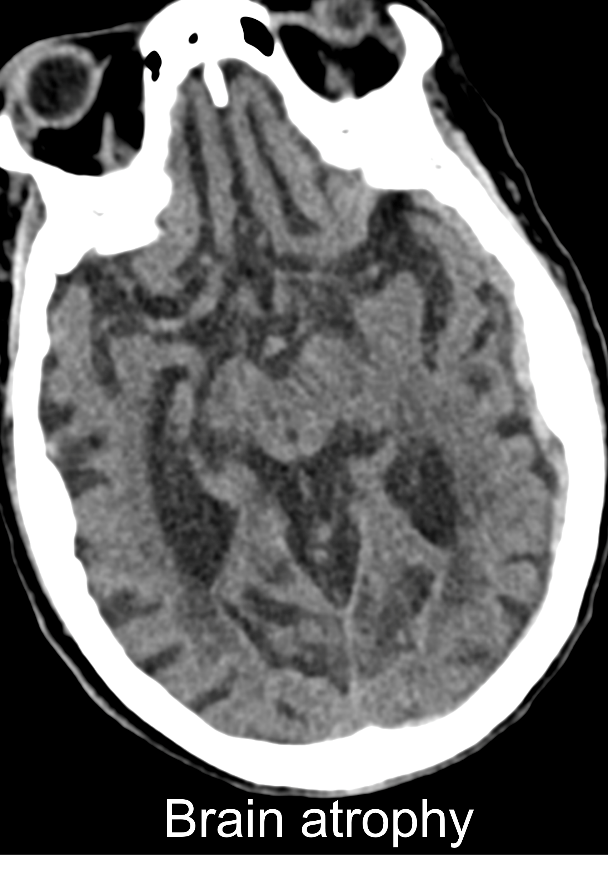
Definition: This entity refers to loss of tissue volume over time due to cell death or shrinkage. When strictly defined, a change should be seen over serial images to confirm that the changes are due to a specific traumatic event, rather than being preexisting. In some cases, atrophy can be inferred at a single time point due to patterns of brain appearance (for example, a smaller size and increased signal of one hippocampus compared to the other or severe atrophy of the posterior cingulate gyrus, precuneus and parietal lobes). It should be noted that enlargement of the subarachnoid spaces does not in itself confirm atrophy, as it may represent primary problems with CSF hydrodynamics (for instance, in infancy or early after traumatic subarachnoid hemorrhage). In addition, it should be noted that atrophy can only be seen either over time or in the context of head circumference as in children.

Appearance on CT: Cortical atrophy manifests itself as gyral volume loss in the affected lobes and with widening the ventricles, sulci, and/or fissures.

Appearance on MRI: Cortical atrophy is best visualized on anatomical images, including T1-weighted, T2-weighted, and/or T2-weighted FLAIR images, and also manifests itself as a general or local widening of the ventricles, sulci, fissures with associated gyral volume loss in the affected lobes. Medial temporal lobe atrophy is best visualized on coronal T1-weighted imaging, and manifests itself as a widening of the choroid fissure, followed by progressive widening of the temporal horn of the lateral ventricle and a decrease in hippocampal volume. Parietal atrophy manifests as a widening of the posterior cingulate, precuneus and parieto-occipital sulci on sagittal T1-weighted and FLAIR imaging.

Core:

Is Present.

Might be Present.

Not Present.

Supplementary:

*Location (check all that apply):*

Frontal

R L

Parietal

R L

Temporal cortex

R L

Hippocampus

R L

Occipital

R L

Deep gray matter

R L

Supratentorial white matter (corpus callosum, periventricular white matter) R L

Cerebellum

R L

Brainstem

Midbrain Pons Medulla

Emerging: Brain volumetric analysis, asymmetry metrics, atrophy patterns of differing pathologies

##
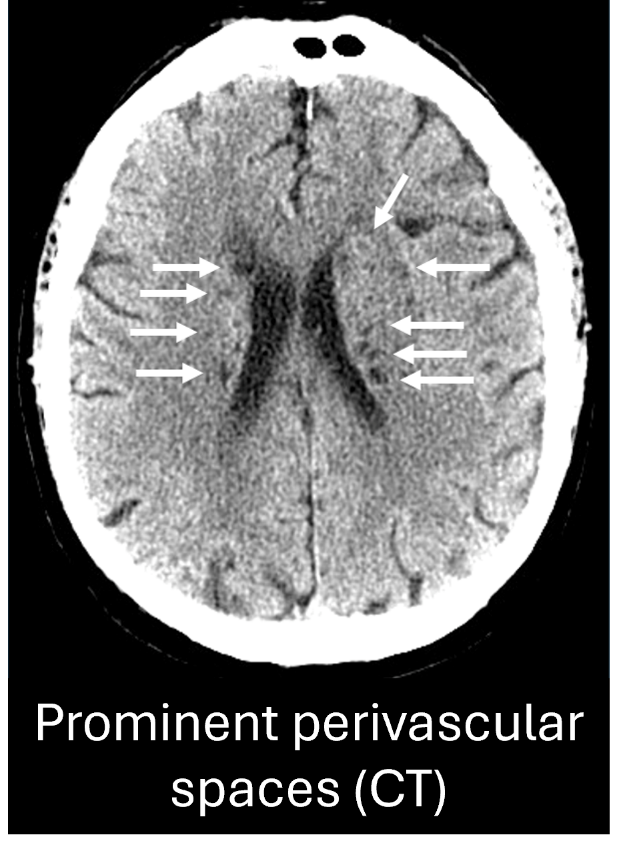
Enlarged Perivascular Spaces

Definition: Perivascular spaces, also referred to as Virchow-Robin spaces, are normal pial-lined spaces filled with CSF or interstitial fluid that surround small perforating blood vessels in the brain. They are believed to play an important role in clearing metabolic waste. When their diameters exceed 1 mm on high-resolution anatomical MRI, they are typically classified as enlarged perivascular spaces (EPVS). They are commonly encountered in the centrum semiovale, basal ganglia, insular region, and anterior temporal pole.

Appearance on CT: EPVS usually appear as oval, round or tubular hypodense areas with a diameter exceeding 1 mm on CT. Distinguishing sizeable EPVS from lacunar infarcts relies on their location and shape. Large EPVS typically exhibit a well-defined, symmetrical shape with smooth margins, and often align with the path of perforating arteries.


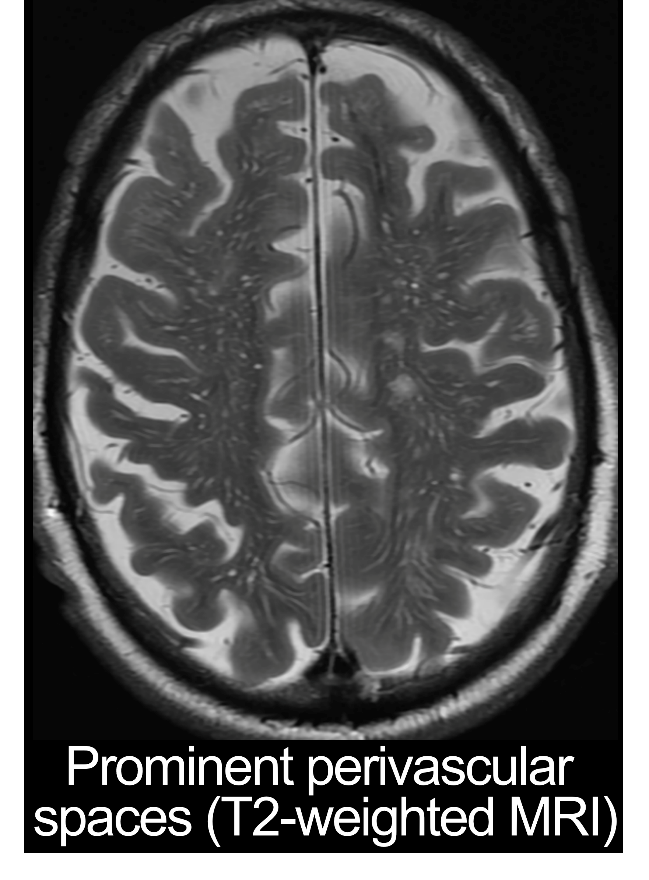
Appearance on MRI: Enlarged perivascular spaces follow CSF signal on all pulse sequences. They are hypointense on T1-weighted imaging, and hyperintense on T2-weighted images with a diameter that is larger than 1 mm. They are typically linear or curvilinear when parallel and ellipsoidal when perpendicular to the imaging plane. As opposed to subcortical infarcts, they don’t show a rim or area of high signal intensity on FLAIR and there is typically no evidence of a hemosiderin rim on T2*-weighted GRE.

Core:

Is Present.

Might be Present.

Not Present.

Supplementary:

*Location (check all that apply):*

Centrum semiovale R L

Basal ganglia R L

Insular/Temporal pole R L

Hippocampus R L

Mesencephalon R L

Emerging: Volumes. The following imaging modalities can be used to evaluate and automatically segment PVS, including DWI, T2-weighted FLAIR, T2-weighted, T1-weighted black-blood, or phase-contrast imaging^14^.

## Other Imaging Findings

## Additional imaging findings, which may include incidental findings, can be documented in a provided free-text space.

# ABBREVIATIONS USED

ADC - apparent diffusion coefficient

ASL – arterial spin labeling

CDE - common data elements

CSF - cerebrospinal fluid

CTA - computed tomography angiography

CT - computed tomography

CTV - computed tomographic venography

DAI - diffusion axonal injury

DCE - dynamic contrast enhanced

DSC - dynamic susceptibility contrast

DWI - diffusion weighted imaging

EDH - epidural hematoma

EMR – electronic medical record

EPVS – enlarged perivascular space

GRE - gradient-recalled echo

GWD – grey-white differentiation

ICP - intracranial pressure

IVH –iIntraventricular hemorrhage

MAP - mean arterial pressure

MCA – middle cerebral artery

MRA - magnetic resonance angiography

MRI - magnetic resonance imaging

MR - magnetic resonance

MRV – magnetic resonance venography

NINDS - National Institute of Neurological Disorders and Stroke

PCA – posterior cerebral artery

QSM - quantitative susceptibility mapping

SAH - subarachnoid hemorrhage

SDH - subdural hematoma

SWAN - Susceptibility-Weighted Angiography

SWI - susceptibility weighted imaging

T – Tesla (magnet strength unit)

T2* - T2 star (T2-weighted GRE)

T2 FLAIR - T2-weighted Fluid Attenuated Inversion Recovery

TAI - traumatic axonal injury

TBI - traumatic brain injury

WM - white matter

**REFERENCES**

1. Duhaime AC, Gean AD, Haacke EM, et al. Common data elements in radiologic imaging of traumatic brain injury. Arch Phys Med Rehabil 2010;91(11):1661-6, doi:10.1016/j.apmr.2010.07.238

2. Steyerberg EW, Mushkudiani N, Perel P, et al. Predicting outcome after traumatic brain injury: development and international validation of prognostic scores based on admission characteristics. PLoS Med 2008;5(8):e165; discussion e165, doi:10.1371/journal.pmed.0050165

3. Yuh EL, Jain S, Sun X, et al. Pathological Computed Tomography Features Associated With Adverse Outcomes After Mild Traumatic Brain Injury: A TRACK-TBI Study With External Validation in CENTER-TBI. JAMA Neurol 2021;78(9):1137-1148, doi:10.1001/jamaneurol.2021.2120

4. Haghbayan H, Boutin A, Laflamme M, et al. The Prognostic Value of MRI in Moderate and Severe Traumatic Brain Injury: A Systematic Review and Meta-Analysis. Crit Care Med 2017;45(12):e1280-e1288, doi:10.1097/CCM.0000000000002731

5. Vande Vyvere T, De La Rosa E, Wilms G, et al. Prognostic Validation of the NINDS Common Data Elements for the Radiologic Reporting of Acute Traumatic Brain Injuries: A CENTER-TBI Study. J Neurotrauma 2020;37(11):1269-1282, doi:10.1089/neu.2019.6710

6. Haydel MJ, Preston CA, Mills TJ, et al. Indications for computed tomography in patients with minor head injury. N Engl J Med 2000;343(2):100-5.

7. Stiell IG, Wells GA, Vandemheen K, et al. The Canadian CT Head Rule for patients with minor head injury. Lancet 2001;357(9266):1391-6, doi:10.1016/s0140-6736(00)04561-x

8. Head injury: assessment and early management. London, UK; 2023. Available from: <https://www.nice.org.uk/guidance/ng232> [Last Accessed; 06-January].

9. Smits M, Dippel DW, Steyerberg EW, et al. Predicting intracranial traumatic findings on computed tomography in patients with minor head injury: the CHIP prediction rule. Ann Intern Med 2007;146(6):397-405, doi:10.7326/0003-4819-146-6-200703200-00004

10. Griffin AD, Turtzo LC, Parikh GY, et al. Traumatic microbleeds suggest vascular injury and predict disability in traumatic brain injury. Brain 2019;142(11):3550-3564, doi:10.1093/brain/awz290

11. Edlow BL, Haynes RL, Takahashi E, et al. Disconnection of the ascending arousal system in traumatic coma. J Neuropathol Exp Neurol 2013;72(6):505-23, doi:10.1097/NEN.0b013e3182945bf6

12. Saver JL, Warach S, Janis S, et al. Standardizing the structure of stroke clinical and epidemiologic research data: the National Institute of Neurological Disorders and Stroke (NINDS) Stroke Common Data Element (CDE) project. Stroke 2012;43(4):967-73, doi:10.1161/STROKEAHA.111.634352

13. Biffl WL, Moore EE, Offner PJ, et al. Blunt carotid arterial injuries: implications of a new grading scale. J Trauma 1999;47(5):845-53, doi:10.1097/00005373-199911000-00004

14. Dubost F, Yilmaz P, Adams H, et al. Enlarged perivascular spaces in brain MRI: Automated quantification in four regions. Neuroimage 2019;185(534-544, doi:10.1016/j.neuroimage.2018.10.026
